# ***Phascolarctobacterium faecium*** **reduces food intake via PYY signaling, contributing to the mitigation of body weight gain in diet-induced obese mice**

**DOI:** 10.1080/19490976.2026.2617691

**Published:** 2026-01-21

**Authors:** Clara Bullich-Vilarrubias, Marina Romaní-Pérez, Inmaculada López-Almela, Carlos Pomares-Díaz, Silvia Basili Franzin, Giuseppe Esposito, Alfonso Benítez-Páez, Verónica Tolosa-Enguís, Yolanda Sanz

**Affiliations:** a Microbiome Innovation in Nutrition & Health Research Unit (INNOBIOME), Institute of Agrochemistry and Food Technology, Spanish National Research Council (IATA-CSIC), Valencia, Spain; b Department of Physiology and Pharmacology, Sapienza University of Rome, Rome, Italy

**Keywords:** Gut microbiota, anorexigenic hormones, PYY, gut transit, obesity, food intake

## Abstract

Excess energy intake contributes to adiposity in obesity. We investigated whether the human intestinal bacterium *Phascolarctobacterium faecium* could prevent obesity *via* enteroendocrine pathways in a mouse model of diet-induced obesity (DIO). Daily administration of *P. faecium* (2 × 10^9^ cells/mouse) reduced food intake through the early overproduction of the satiety hormone peptide YY (PYY) compared to untreated DIO mice. Moreover, *P. faecium* increased the intestinal levels of branched-chain amino acids, which, in turn, stimulated PYY secretion in neuroendocrine cell cultures and also modified gut microbiota composition. A pair-feeding study demonstrated that the anorexigenic effect of *P. faecium* contributes to its effects in attenuating body weight gain in DIO mice, but that other mechanisms are also involved in its metabolic benefits. Specifically, *P. faecium* accelerated gut transit and serum lipid clearance, thereby limiting adiposity independently of food intake. This study identifies the mode of action of a human intestinal bacterium recently linked to obesity protection, providing valuable insights into host-microbe interactions governing body weight.

## Graphical Abstract

**Figure:**
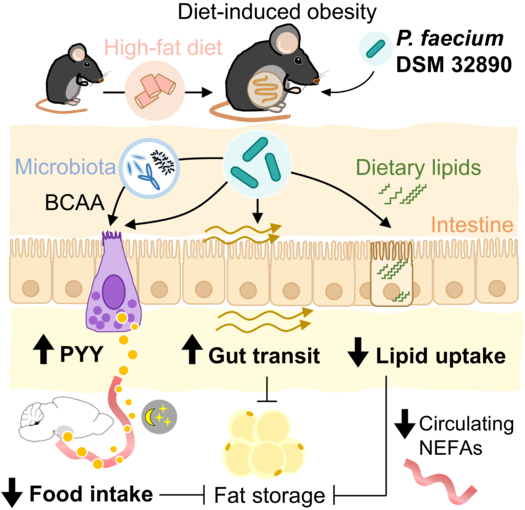

## Introduction

Obesity has reached epidemic proportions,^1^^,^^2^ and its multifactorial etiology complicates the development of effective therapies to curb its growing prevalence. In particular, diets high in calories, saturated fats and simple sugars disrupt energy balance, significantly disrupting hypothalamic brain circuits that govern food intake.^3-9^ The gut also plays an important role in controlling food intake and energy homeostasis by sensing nutrients from digested foods and transmitting this information to the brain. Nutrient sensing is mediated by specialized enteroendocrine cells (EECs) within the gut lining.^10^ EECs produce gut hormones^10^^,^^11^ that communicate with the brain through two primary pathways: (1) endocrine signaling, where hormones are transported through the bloodstream, and (2) paracrine signaling, involving the activation of vagal afferent neurons innervating the intestinal mucosa.^12^^,^^13^

Obesity is linked to altered secretion of anorexigenic gastrointestinal hormones, such as glucagon-like peptide 1 (GLP-1) and peptide YY (PYY), whose levels can be decreased or enhanced likely depending on the prandial phase and obesity progression.^14-19^ Accordingly, obesity treatments are being developed that leverage gut peptide mechanisms. These strategies aim to mimic the enteroendocrine responses observed after bariatric surgery such as enhanced postprandial secretion of GLP-1, PYY, and CCK collectively contributing to weight loss and improved glucose homeostasis.^20^ These approaches include gut hormone analogues^21^^,^^22^ and agonists of G-protein coupled receptors (GPCRs) that stimulate gut hormone secretion (secretagogues), with the latter currently under investigation.^23^ Moreover, substantial evidence indicates that consuming a Western diet alters gut microbiota composition, ^24-27^ contributing to obesity by increasing the amount of energy extracted from food.^28^ Intestinal bacteria also influence host feeding behavior by influencing satiety and reward pathways.^29^^,^^30^ Bacterial components and metabolites generated during food digestion and fermentation can stimulate EECs *via* toll-like receptors (TLRs) and GPCRs, respectively, leading to the synthesis and release of appetite-regulating hormones.^31-34^ For example, nutrient-induced growth of *Escherichia coli* promotes the production of the ClpB protein, which contributes to the sensation of fullness by increasing PYY secretion and activating hypothalamic proopiomelanocortin (POMC) neurons.^35^ Moreover, short-chain fatty acids (SCFAs) such as acetate, propionate, and butyrate, produced by bacterial fermentation of dietary fibres, activate FFAR2/3 receptors on EECs to promote the secretion of GLP-1 and PYY.^36^^,^^37^ Despite the potential of human intestinal bacteria to reduce caloric intake in obesity, direct evidence establishing their causal role remains limited. One observational study spanning four years found that the abundance of the genus *Phascolarctobacterium* decreased in children experiencing excessive body weight gain compared with those with normal weight gain^38^ and *P. faecium* inversely associates with overweight and obesity in a large meta-analysis including more than 7,500 adult human subjects.^39^ Moreover, *Phascolarctobacterium* has been implicated in the antidiabetic effects of metformin or berberine treatment in obese rats.^40^ At species level, caloric restriction in obese individuals led to an increased abundance of *P. faecium* alongside decreases in both body weight and visceral fat.^41^ However, the specific causal role of *P. faecium* in obesity needs further investigation in translational studies.^39^ Here, we investigated whether the strain *P. faecium* DSM 32890 (referred to as *P. faecium*) affects the enteroendocrine system and food intake—a largely unexplored mechanism with potential anti-obesogenic effects in diet-induced obesity (DIO). Utilizing *in vivo* models (hormone blocking and paired-feeding models) and *in vitro* experimental systems, we demonstrated that *P. faecium* exerts anorexigenic effects by enhancing PYY secretion, a key factor in body-weight control. Our findings also indicated that both the bacterium itself and its elevated intestinal metabolites (branched-chain amino acids, BCAAs) act as PYY secretagogues. Notably, we found that *P. faecium* accelerated gut transit time and reduced lipid absorption, independent of its influence on food intake, providing an additional pathway for obesity prevention.

## Material and methods

### Phascolarctobacterium faecium isolation and culture conditions

*P. faecium* DSM 32890 (referred to as *P. faecium*) was isolated from the feces of a healthy volunteer, as described elsewhere.^42^ For *in vivo* experiments, *P. faecium* was grown in modified PYG medium supplemented with sodium succinate (8 g/L) instead of glucose, at 37°C under anaerobiosis for 48 h. Following centrifugation (10000g, 10 min, 4°C) and two washes with phosphate-buffered saline (PBS), the cells were re-suspended in PBS containing 0.05% cysteine and 20% glycerol. The number of viable bacteria (cells/ml) was determined using BD TrucountTM Tubes (Becton Dickinson, Franklin Lakes, NJ) and propidium iodide staining (Sigma-Aldrich) in a BD LSRFortessa flow cytometer (Becton Dickinson).

### Mice, diets and experimental design

C57BL/6 male mice, aged 6-8 weeks (Charles River Laboratories), were housed individually in a controlled environment with constant humidity and temperature (23 ± 2°C). Animals were maintained on a 12-hour light–dark schedule, with lights switched on at 06:00 and off at 18:00. For reference, zeitgeber time (ZT)0 and ZT12 correspond to lights on and lights off, respectively.

Unless otherwise specified, mice had unrestricted access to either a control diet (CD) with 10% kcal from fat and without sucrose (D12450K), or a high-fat, high-sugar diet (HFHSD) with 45% kcal from fat and 21% from sucrose (D12451), both obtained from Ssniff Spezialdiäten GmbH. Sample size, detailed in Figure S1, was based on our prior experience.^43^ Differences in sample sizes across figures are due to the exclusion of samples due to technical issues and/or insufficient material. Mice were weighed and randomly assigned to experimental groups, resulting in similar body weights across groups. All animal experimental procedures performed complied with the European Union 2010/63/EU and the Spanish RD 53/2013. Procedures were reviewed and approved by the ethics committee of the University of Valencia (SCSIE, UV, Spain) and authorized by the Dirección General de Agricultura, Ganadería y Pesca (Generalitat Valenciana; approval IDs: 2017/VSC/PEA/00015, 2019/VSC/PEA/0020, and 2020/VSC/PEA/0022).

We explored the effects of oral supplementation of *P. faecium* on DIO in mice by conducting the experiments detailed below and graphically represented in Figure S1.

*Experiment 1* (Figure S1a): HFHSD-fed mice received a daily oral gavage of *P. faecium* (2 × 10^9^ cells/mouse in PBS plus 0.05% cysteine, 20% glycerol) or vehicle (PBS plus 0.05% cysteine, 20% glycerol) at the beginning of the dark phase (ZT12) throughout the 12-week experimental period, while CD-fed mice received only vehicle. Body weight was measured weekly and 24-h individual food intake was measured after 3, 4 and 12 weeks of HFHSD-feeding by manually weighing the provided and residual food in each cage. At week 4, body weight loss after overnight fasting was estimated in HFHSD-fed mice receiving vehicle or *P. faecium*. After 4, 8 and 12 weeks of HFHSD-feeding, animals were anesthetized with isoflurane following an overnight fasting, and blood samples were obtained through cardiac puncture. Immediately after cervical dislocation, brains and cecal contents were collected and snap-frozen for later processing. Mice euthanized at week 8 were used to prepare whole-mounts of colonic circular muscle myenteric plexus (CMMP) for immunostaining of enteric neurons and glia.

*Experiment 2*: (Figure S1b): For the pair-feeding experiment, mice were fed HFHSD for 12 weeks and received a daily oral dose of vehicle or *P. faecium*, as in Experiment 1. An additional group, also fed HFHSD, was pair-fed over a 24-h period, receiving an amount of food equal to the average daily consumption of the *P. faecium*-treated group from the previous day. The pair-fed group was orally administered with vehicle. Body weight was monitored weekly and food intake was measured daily at ZT12.

Different functional assessments were performed throughout the intervention, including intestinal absorption of lipids, PYY secretion and glycemia in response to an oral nutritional challenge, intestinal transit time and oral glucose tolerance test (OGTT).

*-Intestinal absorption of lipids, PYY secretion assays and postprandial glycemia*: At weeks 4 and 12, 4-h fasted mice received an oral load of intralipid 20% (300 µL per mouse, Sigma) at ZT12, as reported,^44^ and blood was collected from the saphenous vein at 0 (fasting), 20, 120 and 240 min after intralipid administration. NEFAs were measured at 0, 20, 120 and 240 min; triglycerides at 0, 120 and 240 min; and PYY was measured in samples collected at 0 and 20 min. For PYY analysis, intralipid administration and blood sampling were also performed at ZT0 (light phase) to determine the optimal phase of gut hormone secretion. Glycemia was determined in blood from the saphenous vein at fasting and 20 min after intralipid administration using glucose test strips and a Contour XT glucometer (Bayer).

*-Gastrointestinal transit time*: At week 7, intestinal transit time was determined as reported.^45^ Briefly, mice with free access to food were administered an oral gavage of carmine red dissolved in 0.5% methylcellulose in 0.9% PBS at ZT12. The color of fecal pellets of each mouse was examined every 30 min. Intestinal transit time was measured as the time between oral gavage and first appearance of carmine dye in feces.

*-OGTT*: At week 10, an OGTT was carried out as described.^46^

At week 12, overnight-fasted mice were intraperitoneally injected with insulin (1.5 U/kg, NovoRapid FlexPen®, Novo Nordisk). After 15 min, mice were sacrificed by cervical dislocation. Blood glucose levels were measured from the saphenous vein at fasting and after insulin administration. Epididymal white adipose tissue (eWAT), subcutaneous white adipose tissue (subWAT), and brown adipose tissue (BAT) were dissected and weighed. Fragments of eWAT, subWAT and colon were immersed in 10% neutral buffered formaldehyde for fixation and further hematoxylin-eosin staining and immunohistochemistry. Additionally, fragments of subWAT were immediately frozen for gene expression processing.

*Experiment 3*: (Figure S1c): Experiments were conducted in mice fed HFHSD for 4 weeks. We first performed two dose-response studies to validate the immunoneutralization of PYY by administrating a PYY antibody. Subsequently, we performed a PYY-blocking experiment to test whether *P. faecium* requires PYY to exert its anorexigenic effects.

In the first test, we assessed the capacity of an anti-PYY antibody (Phoenix Pharmaceuticals) to increase food intake. Two concentrations were tested: a dose 10 and 100 times higher than the PYY plasma levels in *P. faecium*-treated mice (i.e., 1500 pg/mouse or 15000 pg/mouse, respectively). Mice were intraperitoneally injected with saline, 10 × dose or 100 × dose of anti-PYY antibody, 1 h before the onset of the dark phase (ZT11). *Ad libitum* food intake was measured at ZT24 for two consecutive days. In the second test, we determined whether the lower effective dose of the anti-PYY antibody could block the anorexigenic effect of exogenously administered PYY. Mice were intraperitoneally injected: saline at ZT11 and at ZT12 (day 1 and day 2); saline followed by 5 µg/kg of PYY_3-36_ (Tocris™ Bioscience) at ZT11 and ZT12, respectively (day 3 and day 4); and a 10 × anti-PYY antibody dose followed by PYY_3-36_ at ZT11 and ZT12, respectively (day 5 and day 6). *Ad libitum* food intake was measured 4 h after the saline/saline, saline/PYY and anti-PYY antibody/PYY_3-36_ administrations (ZT16) for two consecutive days. For the PYY-blocking experiment, mice received a daily oral gavage of *P. faecium* at the same dose as in Experiment 1 or vehicle during 4 weeks. At the end of the intervention, mice were injected intraperitoneally (at ZT11) with saline and then with 10 × anti-PYY antibody, for two consecutive days each. *Ad libitum* food intake was determined after 24 h.

For all experiments, blood samples were collected in Microvette® 500 K3E tubes (Sarstedt). For GLP-1 analysis, blood tubes contained dipeptidyl-peptidase (DPP)-4 inhibitor (Sigma). Plasma was obtained by immediate centrifugation and stored at –80°C until analysis.

### Hormone measurements

Plasma levels of total PYY, total GLP-1 and insulin were quantified using a MILLIPLEX Mouse Metabolic Hormone Expanded Panel (MMHE-44K) on a Luminex® MAGPIX System (Milliplex, Merck). Leptin plasma levels were determined with Mouse Leptin ELISA (Merck), on a CLARIOstar Microplate Reader (BMG Labtech). Mouse Insulin ELISA (Mercodia) was used to measure insulin at 0 and 15 min of OGTT. Hormone concentrations fall within the expected range of the commercial kits used, and absolute values may differ from those obtained with other assay platforms.

### Triglycerides, cholesterol, NEFAs and citrate synthase measurements

Triglycerides, cholesterol and non-esterified fatty acids (NEFAs) were measured in plasma using the Triglyceride Colorimetric Assay Kit (Elabscience) or the Triglyceride Quantification Colorimetric Assay Kit (Abcam), Cholesterol Liquid Kit (Química Clínica Aplicada SA), and Free Fatty Acid Colorimetric Assay Kit (Abcam). Citrate synthase activity was measured in subWAT using the Citrate Synthase Assay Kit (Abcam).

### Acute PYY secretion assay in STC-1 cell line

Enteroendocrine murine STC-1 cells (ATCC®) were grown in Dulbecco's Modified Eagle's Medium (DMEM; ATCC®) supplemented with 10% heat-inactivated fetal bovine serum (FBS) (Gibco™), 1% penicillin/streptomycin (Sigma) and 0.1% amphotericin B (Corning®) in 75 cm^2^ cell culture flasks (SPL Life Sciences) and maintained in an incubator at 37°C in a 5% CO_2_ atmosphere. Cells were then seeded in 24-well cell culture plates (SPL Life Sciences) at a density of 1 × 10^5^ cells per well and incubated at 37ºC for 48 h until reaching 80% confluence. The medium was removed and cells were washed with PBS. Cells, maintained in DMEM, were preincubated with pasteurized *P. faecium* (cell-to-bacteria ratio of 1:10, 50 µl) or an equivalent volume of PBS. After 15 h, PBS-preincubated cells, where further incubated for 1 h with PBS (used as control), the BCAAs valine, leucine, and isoleucine (100 µM each, Sigma), 3-isobutyl-1-methylxanthine (IBMX, 10 µM, Sigma) or forskolin (Fsk, 10 µM, Sigma), used as positive controls. After 16 h (preincubation + incubation), supernatants and cells were harvested and processed to explore the sustained PYY secretory response (induced by 16 h of *P. faecium* incubation) and the acute PYY secretory response (induced by 1 h of BCAAs incubation). Protein from supernatants was concentrated using Amicon® ultrafiltration tubes (0.5 mL) (Merck Millipore). Cells were removed by adding Trypsin-EDTA Solution (ATCC®) and lysed with RIPA buffer (Sigma) with protease inhibitor (Roche). Isolated proteins were stored at before PYY determination using the Mouse PYY EIA kit (RayBiotech).

### Lipid secretion assays in Caco-2 cells

Human colonic Caco-2 cells (ATCC®) were grown in Eagle’s Minimum Essential Medium (EMEM; ATCC®) supplemented with 10% heat-inactivated FBS (Gibco™), 1% penicillin/streptomycin (Sigma) and 0.1% amphotericin B (Corning®) and maintained in a 5% CO_2_ humidified atmosphere at 37°C. Cultured Caco-2 cells were seeded in 12-well Corning® Transwell® plates with polycarbonate membrane inserts (12 mm diameter, 1.12 cm^2^, 0.4 μm pores; Corning®) at a density of 1 × 10^5^ cells per insert. A lipid secretion assay was conducted when cell monolayers reached a trans-epithelial electrical resistance (TEER) > 600 *Ω* x cm^2^, measured with a Millicell-ERS-2 voltohmmeter (Merck-Millipore).

*P. faecium* (cell-to-bacteria 1:10 ratio) or PBS, used as a control, was added to cells of the apical compartment and incubated for 16 h. After one wash with PBS, medium containing lipid micelles (2 mM sodium taurocholate, 0.05 mM cholesterol, 0.6 mM oleic acid, 0.2 mM 2-mono-oleoylglycerol, 0.2 mM L-*α*-lysophosphatidylcholine [Sigma] and 0.02 mM of the fluorescent fatty acid BODIPY™ FL C_16_ [Thermofisher]) was added to the apical compartment for 15 min. Then, medium containing micelles was replaced by fresh medium. BODIPY™ FL C_16_ secretion was determined by measuring fluorescence of both the apical and basal compartments at 15, 120 and 240 minutes after removal of micelles using a CLARIOstar Microplate Reader (BMG Labtech). At 240 min, cells were collected using Trypsin-EDTA Solution (ATCC®), lysed with 0.1% Triton^TM^ X-100 solution and sonicated in an ultrasonic bath for 15 minutes. Lysed cells were centrifuged at 16000g for 3 min at 4°C and supernatants were collected for immediate fluorescence measurements.

### Metabolomic analyses in cecal content

Bile acids (BAs), short-chain fatty acids (SCFAs) and branched amino acids (BCAAs) were analyzed in the cecal content of mice from Experiment 1 according to standardized protocols of LEITAT Technological Center (Valencia, Spain). In brief, samples were extracted in a PreCellys system for BCAAs quantification by ultra-performance liquid chromatography (UPLC) following AccQ-Tag chemical derivatization. SCFAs were analyzed by gas chromatography-mass spectrometry (GC-MS) and the concentration of BAs was determined by UPLC.

### Histology, immunohistochemistry and immunofluorescence

Fixed eWAT, subWAT and 3-cm proximal colon tissues were embedded in paraffin. Following standardized protocols by Patologika Laboratorio S.L. (Valencia, Spain), WAT samples were stained with hematoxylin and eosin, and intestinal samples underwent immunohistochemical staining for quantification of PYY-positive cells using a 1:9000-dilution of anti-PYY antibody (Abcam, Cat. #ab22663) and a specific horseradish peroxidase-conjugated secondary antibody. Bright-field digital images were captured with an Eclipse 90i Nikon wide-field microscope (Nikon Corporation) coupled to a CFI Plan Fluor DIC M/N2 (MRH00200) Nikon dry-air objective of 20 × combined with an optical zoom factor of 0.8 × for adipose tissue samples and 4 × for intestinal samples. Individual adipocyte areas were selected using Fiji software (ImageJ 1.49q Software, National Institutes of Health, USA) and quantified by Nis elements BR 3.2 software (Nikon Corporation). Data are based on measurements of 70-300 adipocytes from 4-6 sections per mouse. For intestinal samples, PYY-positive cells and mucosal areas were selected and quantified using Nis elements BR 3.2 software, and the number of PYY-positive cells was calculated per unit of mucosal area.

Immunostaining of enteric neurons and glia in colon was conducted in CMMP whole-mount preparations obtained as previously described.^47^ Whole-mount preparations were fixed in ice-cold 4% PFA, washed with 1X PBS containing 0.1% Triton X-100 (T-PBS), and incubated for 45 minutes at room temperature in blocking solution (4% normal donkey serum, 0.1% Triton X-100, 1% BSA in PBS). Samples were then treated overnight at 4°C with rabbit anti-GFAP (1:200 v/v, Bioss) and mouse anti-peripherin (1:200 v/v, Santa Cruz Biotechnology). Secondary antibodies (1:400, v/v) donkey anti-rabbit Alexa Fluor 564 (ThermoFisher Scientific) and donkey anti-mouse Alexa Fluor 488 (Jackson Immuno Research) were applied for 2 h at room temperature. Washes in 1X PBS were conducted after each incubation. Sections were mounted in ProLong Diamond Antifade mountant (ThermoFisher Scientific). Images were acquired through the × 20 objective on a Zeiss LSM 980 confocal microscope (SCSIE-UV). Images were anlayzed using Fiji software (ImageJ 1.49q Software, National Institutes of Health, USA) to quantify the average relative fluorescence units (RFU) from 15–20 tissue cross-sections or to determine the number of peripherin-positive neurons per mm^2^.

### RNA extraction and RT-qPCR analysis

For total RNA extraction of hypothalamus and subWAT, TRIsure™ reagent (Bioline) was used according to the manufacturer’s instructions. For reverse transcription, between 1-2 μg of total RNA were incubated at 25°C for 10 min, 37°C for 120 min and 85°C for 5 min with the High-Capacity cDNA Reverse Transcription Kit (Thermo Fisher Scientific) in an Eppendorf thermocycler. Real-time qPCR was carried out by incubating previously validated cDNA dilutions with 300 nM of gene-specific primer pair sequences (Table S1, Isogen) and LightCycler 480 SYBR Green I Master Mix (Roche) in a LightCycler® 480 Instrument (Roche). Relative mRNA levels were calculated for each gene according to the 2^–(ΔΔCt)^ method. The amount of target gene, normalized to the housekeeping gene ribosomal protein L19 (*Rpl19*), is represented as mRNA fold-change expression relative to the control group.

### Fecal microbiota analysis

Microbiota analysis was conducted as previously described.^48^^,^^49^ Briefly, fecal DNA was isolated using the QIAamp PowerFecal DNA kit (Qiagen). The V3–V4 variable regions of the bacterial 16S ribosomal RNA (rRNA) gene were amplified from fecal DNA by PCR, and products were purified, quantified and pooled in equimolar quantities and sequenced with a 2 × 300 PE configuration in the Illumina MiSeq platform (Eurofins Genomics GmbH). Raw data was downloaded and processed for pair-end assembly,^50^ de-multiplexing,^51^ and chimera removal,^52^ based on a reference dataset (SILVA database, Release 138).^53^ Operational Taxonomic Unit (OTU) approach was implemented from rarefied dataset (10,000 sequences per sample, randomly selected) and following reference-free methods.^54^ Alpha diversity descriptors were computed via QIIME v1.9.1.^55^ allowing calculation of phylogenetic distances.^56^ The assessment of community structure across groups was carried out by distance-based redundancy analysis (dbRDA). For taxonomy assessment, we used the full denoised dataset and compositional microbiota-derived (centered log-ratio - CLR) data with prior execution of zero imputation (Bayesian-Multiplicative replacement). The taxonomic identification of OTUs was supported by SINA aligner^57^ and SILVA database (Release 138).

### Statistical analysis

Statistics were performed with GraphPad Prism version 9.5.1 for Windows (GraphPad Software). Normality and equality of variances of data were assessed through the D’Agostino & Pearson test and Bartlett’s test, respectively. One-way analysis of variance (ANOVA) followed by Tukey’s *post hoc* test was conducted to compare three independent groups, either CD-Veh, HFHSD-Veh and HFHSD-*P. faecium* or HFHSD-Veh, HFHSD-*P. faecium* and HFHSD-Veh paired groups. The Kruskal-Wallis test followed by Dunn’s test for multiple comparisons was used for nonparametric data. Two or three-way ANOVA were used to assess the main effects and interactions between variables. When interactions were identified, Tukey’s *post hoc* tests were conducted. Differences were considered significant when *p*-values were *p* < 0.05. Graphs were plotted using GraphPad Prism version 9.5.1. Statistical analyses on microbiota data were conducted in R (version 4.1.2), employing non-parametric methods, such as the Kruskal-Wallis test and pairwise Wilcoxon Rank Sum test for unpaired samples. To account for multiple comparisons among alpha diversity descriptors, Benjamini-Hochberg post hoc correction was applied. Statistical robustness for differences of the microbial community structure, evaluated through interpretative approaches, was completed using the permutation-based adonis function from the vegan package. Differences in OTU abundance between groups were determined using the Kruskal-Wallis test, followed by Benjamini-Hochberg correction. OTUs displaying highly divergent abundance were selected when the Kruskal-Wallis corrected *p*-value was ≤ 0.01. Correlations were determined using Kendall rank correlations. GraphPad Prism version 9.5.1, and *ggplot2* and grid R v4.1.2 packages were used to generate graphs and plots. Heatmap hierarchical clustering of OTUs (scaled DNA read counts) was achieved using Euclidean distance and the complete clustering method.

## Results

### P. faecium reduces food intake in diet-induced obese mice by amplifying the production and secretion of PYY

**Figure 1.**
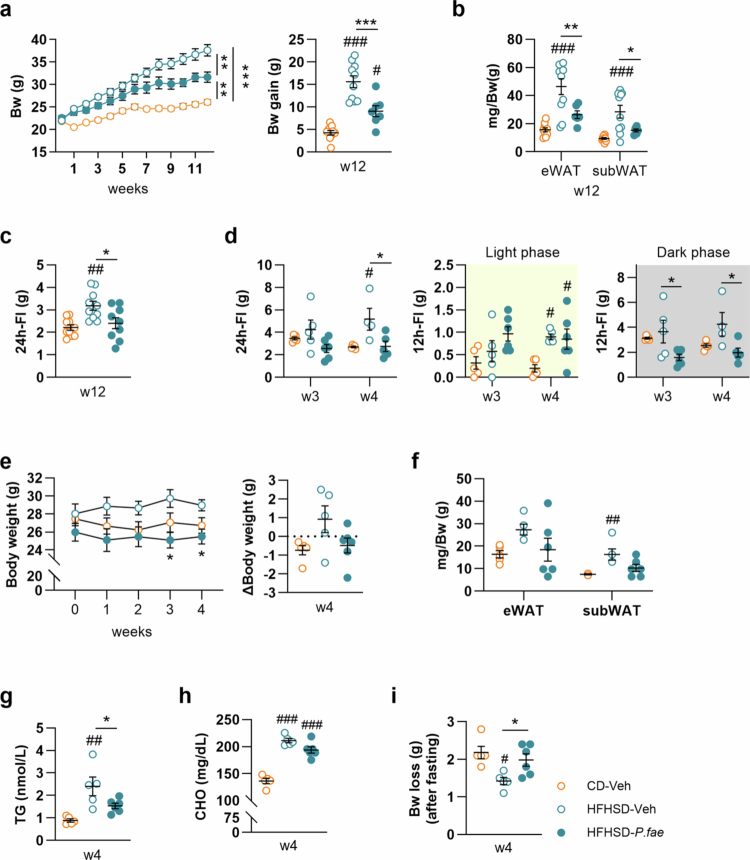
*P. faecium* improves the metabolic phenotype of diet-induced obese mice and reduces food intake. In mice fed CD or HFHSD treated with Veh or with *P. fae*, we measured: (a) Body weight (Bw) follow-up and Bw gain, (b) eWAT and subWAT weight normalized by body weight, and (c) 24 h-FI after 12 weeks of intervention. (d) 24 h- and 12 h- (light and dark phase) FI after 3 and 4 weeks of intervention. (e) Bw evolution and variations (Δ Body weight) after 4 weeks of intervention. (f) Weight of eWAT and subWAT normalized by body weight, after 4 weeks of intervention. Plasma levels of (g) TG and (h) CHO after 4 weeks of intervention. (i) Bw loss after an overnight fast, after 4 weeks of intervention. Abbreviations: Bw: body weight; CD: control diet; CHO: cholesterol; eWAT: epididymal white adipose tissue; FI: food intake; HFHSD: high-fat, high-sugar diet; *P. fae*: *Phascolarctobacterium faecium*; subWAT: subcutaneous white adipose tissue; TG: triglycerides; Veh: vehicle; w- week. a−c (12-week intervention): CD/HFHSD-Veh *n* = 10 per group and HFHSD-*P. fae* *n* = 6−9; d-i (4-week intervention): CD/HFHSD-Veh *n* = 5 per group and HFHSD-*P. fae* *n* = 6. HFHSD-*P. fae* measurements were excluded due to invalid values in Figure 1a (BW—3 data points excluded due to mouse aggression), Figure 1b (adiposity—4 data points excluded due to inadequate sample collection), and Figure 1c (FI—1 data point excluded due to food spillage). Data shown as mean ± SEM. One-way ANOVA followed by Tukey’s *post-hoc* test (a-body weight gain, b, c, e**-**ΔBw gain, d, f−i); two-way ANOVA followed by Tukey’s *post-hoc* test (a and e [body weight evolution]). Significant effects at ^#^*p* < 0.05, ^##^*p* < 0.01 and ^###^*p* < 0.001 versus CD-Veh group; *p < 0.05, **p < 0.01 and ***p < 0.001.

Oral administration of *P. faecium* to mice fed HFHSD for 12 weeks led to a significant decrease in body weight gain (Figure 1a, *p* < 0.001) accompanied by lower weights of eWAT (Figure 1b, *p* = 0.019) and subWAT adipose tissue (Figure 1b, *p* = 0.050). Twenty-four-hour food intake was significantly higher in untreated DIO mice than in CD-fed mice (Figure 1c, *p* = 0.003), and this was reduced by *P. faecium* administration, reaching levels comparable with CD-fed mice after the 12-week intervention (Figure 1c, *p* = 0.020). To determine when *P. faecium* started to suppress food intake during HFHSD feeding, we measured food intake weekly in a separate experiment. *P. faecium* administration prevented the development of hyperphagia in HFHSD-fed mice at 4 weeks, but not at 3 weeks (Figure 1d, CD-Veh vs HFHSD-Veh, *p* = 0.014; HFHSD-Veh vs HFHSD-*P. fae*, *p* = 0.016). Specifically, food intake suppression was observed during the dark phase, but not the light phase, after 12 hours of *ad libitum* feeding (Figure 1d: week 3, *p* = 0.019; week 4, *p* = 0.019).

We next investigated whether the anorexigenic effects of *P. faecium* in the early stages of DIO correlated with metabolic improvements. *P. faecium* treatment reduced body weight during the 3- and 4-week administration periods (Figure 1e: week 3, *p* = 0.017; week 4, *p* = 0.020), although there were no significant net differences in body weight change at the end of the 4-week intervention (Δ Body weight), except for a tendency of *P. faecium* to prevent weight gain compared to untreated HFHSD-fed mice (t-test, *p* = 0.060). Compared to CD-fed mice, untreated HFHSD-fed mice showed an increase in subWAT weight (Figure 1f: CD-Veh vs HFHSD-Veh, *p* = 0.009), whereas this effect was not observed in *P. faecium*-treated mice (Figure 1f). No significant differences were detected in eWAT weight among the groups (Figure 1f). Similarly, after 4 weeks of intervention, the elevated plasma triglyceride levels observed in untreated HFHSD-fed mice **(**Figure 1g: CD-Veh vs HFHSD-Veh, *p* = 0.022) were reduced by *P. faecium* treatment (Figure 1g: HFHSD-Veh vs HFHSD-*P. fae*, *p* = 0.054). However, cholesterol levels remained elevated in both untreated and *P. faecium*-treated HFHSD-fed mice (Figure 1h). Furthermore, *P. faecium* administration resulted in significantly greater body weight loss following a 12-hour fast compared to untreated HFHSD-fed mice (Figure 1i: CD-Veh vs HFHSD-Veh, *p* = 0.010, and HFHSD-Veh vs HFHSD-*P. fae*, *p* = 0.045), suggesting that the bacterium enhanced the utilization of energy stores, likely from adipose tissue, during fasting in the early stages of DIO.

**Figure 2.**
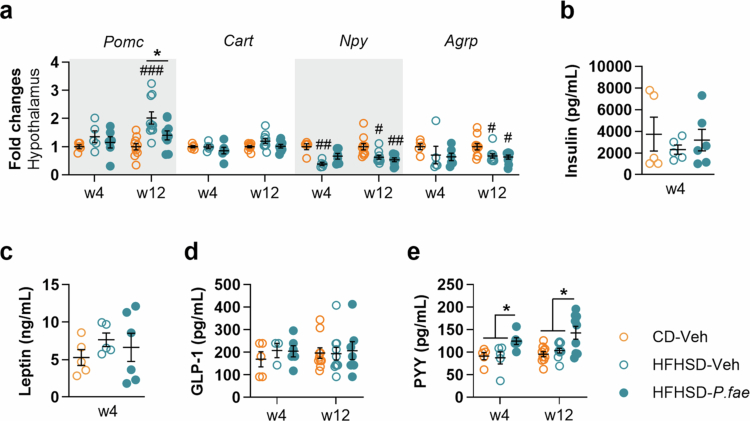
*P. faecium* increases the plasma levels of the anorexigenic hormone PYY in diet-induced obese mice. In mice fed CD or HFHSD treated with Veh or with *P. fae*, we measured: (a) Gene expression of anorexigenic (*Pomc* and *Cart*) and orexigenic (*Npy* and *Agrp*) neuropeptides in the hypothalamus after 4 and 12 weeks of intervention. (b) Plasma levels of insulin and (c) leptin after 4 weeks of intervention. (d) Plasma levels of GLP-1 and (e) PYY after 4 and 12 weeks of intervention. Abbreviations: CD: control diet; HFHSD: high-fat, high-sugar diet; *P. fae*: *Phascolarctobacterium faecium*; Veh: vehicle; w- week. 12-week intervention: CD/HFHSD-Veh *n* = 10 per group and HFHSD-*P. fae* *n* = 9-10; 4-week intervention: CD/HFHSD-Veh *n* = 4-5 per group and HFHSD-*P. fae* *n* = 5-6. Measurements were excluded due to invalid values in Figure 2a (gene expression of hypothalamic neuropeptides—1 data point excluded from HFHSD-*P. fae* due insufficient RNA material), Figure 2d (GLP-1—1 and 3 data points from HFHSD-Veh and HFHSD-*P. fae*, respectively, excluded due to levels below the detection range), and Figure 2e (PYY—1 data point from HFHSD-Veh and HFHSD-*P. fae* due to levels below the detection range). Data shown as mean ± SEM. One-way ANOVA followed by Tukey’s *post-hoc* test. Significant effects at ^#^*p* < 0.05, ^##^*p* < 0.01 and ^###^*p* < 0.001 versus CD-Veh group; *p < 0.05. See also Table S1.

Given these findings, we analyzed the gene expression of hypothalamic neuropeptides involved in the central control of food intake at weeks 4 and 12 of HFHSD feeding. Analysis of anorexigenic neuropeptide mRNA levels revealed that *Pomc* gene expression was significantly higher in untreated HFHSD-fed mice than in CD-fed mice, but only at the end of the intervention (week 12, Figure 2a: CD-Veh vs HFHSD-Veh, *p* < 0.001), and this was normalized by *P. faecium* administration (Figure 2a: HFHSD-Veh vs HFHSD-*P*. *fae*, *p* = 0.047). By contrast, *Cart* expression was unaffected by HFHSD feeding or *P. faecium* treatment (Figure 2a). Regarding orexigenic neuropeptides, the mRNA levels of *Npy* were significantly lower in untreated HFHSD-fed mice than in CD-fed mice at both 4 and 12 weeks of HFHSD feeding (Figure 2a: week 4, *p* = 0.002 and week 12, *p* = 0.011). Lower expression of *Npy* was also found after 12 weeks of *P. faecium* treatment, but not after 4 weeks (Figure 2a, *p* = 0.002). The transcript levels of the orexigenic neuropeptide *Agrp* mirrored the *Npy* gene expression profile, showing reductions only after 12 weeks of HFHSD feeding, irrespective of whether the mice were treated or not with *P. faecium* (Figure 2a: CD-Veh vs HFHSD-Veh, *p* = 0.037 and CD-Veh vs HFHSD-*P*. f*ae*, *p* = 0.020).

To investigate how *P. faecium* might reduce appetite in DIO mice, we measured plasma levels of hormones involved in energy intake regulation. Insulin and leptin, which play a role in long-term energy balance, were not significantly altered by either HFHSD or *P. faecium* treatment after 4 weeks (Figure 2b and 2c). We also measured the anorexigenic hormones GLP-1 and PYY, which are postprandially secreted by EECs, after 4 and 12 weeks of HFHSD feeding. *P. faecium* treatment did not change GLP-1 levels in DIO mice at either time point (Figure 2d), but it significantly increased plasma PYY levels at both 4 and 12 weeks of HFHSD feeding (Figure 2e: HFHSD-Veh vs HFHSD-*P. fae* week 4, *p* = 0.047; week 12, *p* = 0.019), whereas PYY levels in untreated HFHSD-fed mice were similar to those in CD-fed mice (Figure 2e).

Taken together, these data suggest that *P. faecium* administration induces an appetite-reducing effect from the early stages of DIO, potentially through increased PYY secretion. This early hormonal change might precede the longer-term metabolic benefits observed on body weight and adiposity.

**Figure 3.**
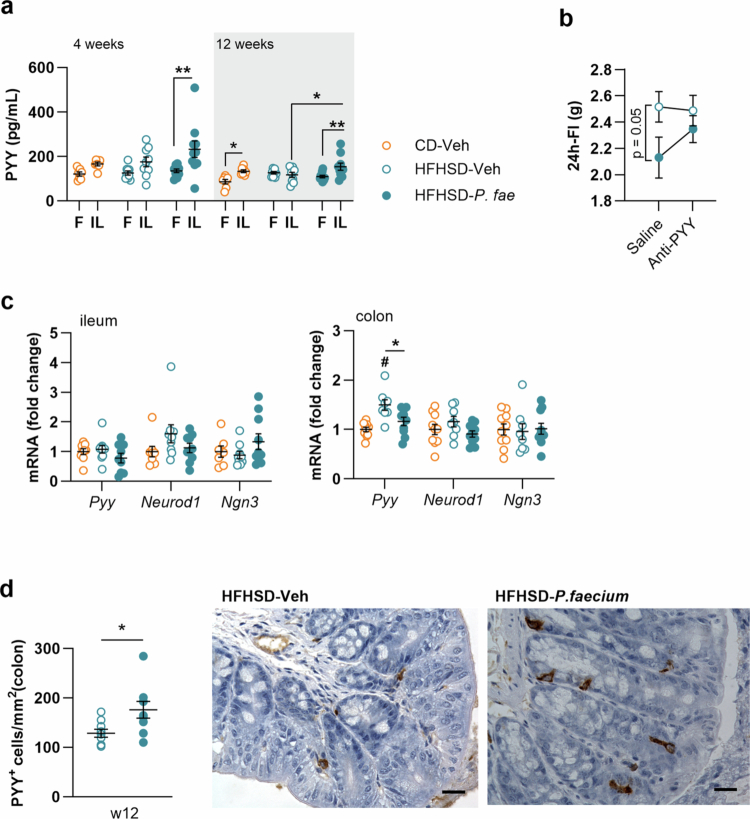
*P. faecium* increases PYY levels and the number of PYY-producing cells after an intralipid oral load in diet-induced obese mice, and requires PYY to suppress food intake. In mice fed CD or HFHSD treated with Veh or with *P. fae*, we measured PYY levels in plasma at fasting and 20 min after an oral load of intralipid 20% at the beginning of dark phase after 4 and 12 (a) weeks of the intervention. (b) 24 h-FI 1 h after intraperitoneal administration of PYY antibody after 4 weeks of intervention. (c) Gene expression of *Pyy*, *Neurod1* and *Nng3* in ileum and colon (d) Quantification of PYY-positive cells in colon and representative bright field images of PYY immunohistochemical staining at 40 × magnification (scale bar = 20 μm). Abbreviations: F: fasting; FI: food intake; HFHSD: high-fat, high-sugar diet; IL: 20 min after intralipid; *P. fae*: *Phascolarctobacterium faecium*; Veh: vehicle; w- week. CD-Veh *n* = 7−10 and HFHSD-Veh/*P. fae* *n* = 8-10 per group. Measurements were excluded due to invalid values in Figure 3a (PYY levels—3 and 1 data points excluded from CD/HFHSD-veh, respectively, due to levels below the detection range), Figure 3b (FI—3 and 1 data points after saline or PYY antibody injections, respectively, from HFHSD-Veh and HFHSD-*P. fae*), Figure 3c (gene expression values—3 data points in the ileum and 2 data points in the colon due to insufficient RNA material). Data shown as mean ± SEM. Two-way ANOVA followed by Tukey´s *post-hoc* test (a and b); One-way ANOVA followed by Tukey´s *post-hoc* test (**c**); Student´s t-test (d). Significant effects at ^#^*p* < 0.05 versus CD-Veh group and *p < 0.05 and **p < 0.01. See also Figure S2.

To further explore how *P. faecium* might stimulate PYY, we examined PYY secretion in response to a nutritional challenge of a 20% lipid emulsion (intralipid). We first determined the optimal phase of the light-dark cycle to observe PYY secretion after intralipid administration in both untreated and *P. faecium*-treated mice after 4 weeks of HFHSD feeding. We observed that PYY plasma levels were influenced by the treatment (vehicle/*P. faecium*), the feeding state (fasting/intralipid) and the daily phase (light/dark). The highest PYY levels were found in *P. faecium*-treated mice after intralipid administration during the dark phase (Figure S2a). *Post-hoc* analyses confirmed that postprandial PYY levels in *P. faecium*-treated mice were significantly higher in the dark phase than in the light phase (Figure S2a). Based on these findings, we conducted the secretion assay in the dark phase after 4 and 12 weeks of HFHSD feeding. After 4 weeks, a significant main effect of nutritional status (fasting vs. intralipid; *p* < 0.001) was observed (Figure 3a), indicating elevated PYY levels following intralipid administration. *Post-hoc* analysis indicated that, in response to intralipid, PYY levels were particularly increased in *P. faecium*-treated mice, which tended to exhibit higher PYY concentrations compared to untreated HFHSD-fed mice (Figure 3a; *p* = 0.080). On the other hand, after 12 weeks of HFHSD feeding, an interaction between the nutritional status and the treatment was found (Figure 3a, *p* = 0.027). *Post-hoc* analysis showed that *P. faecium*-treated mice had higher circulating PYY levels after intralipid administration compared to untreated mice (Figure 3a, *p* = 0.032). Additionally, intralipid administration induced PYY secretion in CD-fed mice and *P. faecium*-treated mice, not in untreated mice, when compared to fasting levels (Figure 3a: Fasting vs Intralipid, *p* = 0.012 for CD-Veh and *p* = 0.006 for HFHSD-*P. fae*), highlighting a benefit of *P. faecium* in maintaining proper gut hormone responses in the context of prolonged HFHSD feeding.

As these data further support that *P. faecium* enhances PYY secretion in DIO mice over both short and long durations, we next investigated whether blocking PYY could reverse the food intake-suppressing effects of *P. faecium*. We first tested the effectiveness of PYY immunoneutralization by intraperitoneally injecting an IgG antibody against PYY and measuring food intake. In a dose-response experiment, we found that HFHSD-fed mice pretreated with a 10 × concentration of anti-PYY antibody, but not a 100 × concentration, showed a significant increase in 12-hour food intake in the dark phase compared with control mice receiving saline (Figure S2b). Furthermore, in a subsequent 4-week preliminary experiment to confirm antibody efficacy, we found that a 10 × dose of the anti-PYY antibody significantly attenuated the reduction in food intake caused by exogenous PYY administration in HFHSD-fed mice (Figure S2c). Based on these findings, we then measured food intake after 4 weeks of HFHSD feeding in both untreated and *P. faecium*-treated mice that received a 10 × dose of the anti-PYY antibody. Consistent with earlier results (Figure 1c), *P. faecium* administration decreased food intake in HFHSD-fed mice receiving saline compared with untreated HFHSD-fed mice (Figure 3b, *p* = 0.051). However, this reduction in food intake was not observed when mice were administered simultaneously the PYY antibody (Figure 3b).

To elucidate whether *P. faecium* also affects PYY production in the gut, we first examined ileal and colonic gene expression of *Pyy* and the transcription factors *Neurod1* and *Neurogenin 3*, which play essential roles in the development and differentiation of EECs. In the colon, transcript levels of *Pyy* were elevated in untreated HFHSD-fed mice (Figure 3c: CD-Veh vs HFHSD-Veh, *p* < 0.001), and *P. faecium* restored these levels to those of CD-fed mice (Figure 3c: HFHSD-Veh vs HFHSD-*P. fae*, *p* = 0.019). No significant differences were detected in the expression of transcription factors in the colon; likewise, these genes remained unaffected by either the HFHSD or the bacterial treatment in the ileum (Figure 3c).

Finally, immunohistochemical analysis of the colon revealed that the density of PYY-positive cells per unit of mucosal area was significantly greater in *P. faecium*-treated mice than in untreated mice on HFHSD (Figure 3d, *p* = 0.027). This apparent divergence between *Pyy* mRNA expression and PYY protein levels is biologically plausible as additional post-transcriptional and post-secretory regulatory processes may occur. All in all, our results indicate that *P. faecium* increases both the number of PYY-producing EECs and the secretion of PYY, and this PYY hypersecretion is likely required for reducing food intake in DIO mice treated with *P. faecium*.

### P. faecium drives changes in gut microbiota composition and derived metabolites

**Figure 4.**
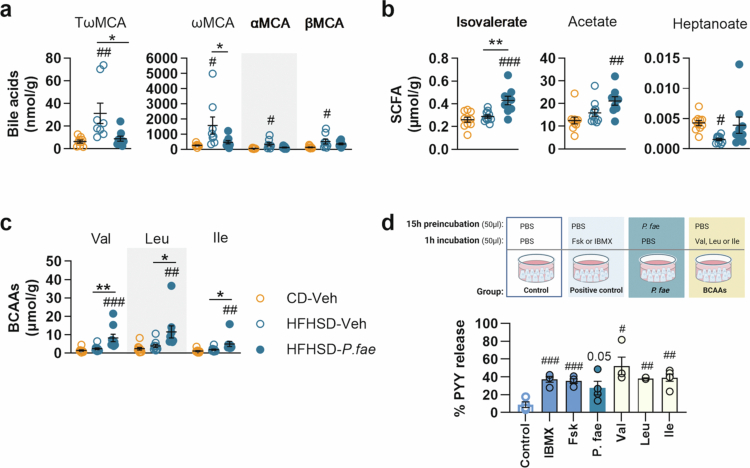
*P. faecium* increases the cecal levels of branched-chain amino acids, which act as PYY secretagogues in SCT-1 neuroendocrine cells. Levels of (a) bile acids, (b) SCFAs and (c) BCAAs in the cecum content of mice fed CD or HFHSD treated with Veh or with *P. fae*, after 12 weeks of intervention. (d) Percentage of PYY secretion *in vitro* by STC-1 cells incubated with PBS (control conditions), *P. fae* or BCAAs. Abbreviations: αMCA: *α*-muricholic acid; βMCA: *β*-muricholic acid, ωMCA: *ω*-muricholic acid; BCAAs: branched-chain amino acids; CD: control diet; HFHSD: high-fat, high-sugar diet; Ile: isoleucine; Leu: leucine; Val: valine; *P. fae*: *Phascolarctobacterium faecium*; SCFAs: short-chain fatty acids; TωMCA: tauro-*ω*-muricholic acid; Veh: vehicle. **a−c**: CD-Veh *n* = 9-10, HFHSD-Veh *n* = 8−10 and HFHSD-*P. fae* *n* = 9; d: *n* = 4 for all conditions, except Leu (*n* = 3) and Ile (*n* = 5). Measurements were excluded due to invalid values in Figure 4a (bile acids—1 data point excluded from CD-Veh and HFHSD-*P. fae* and 2 data points excluded from HFHSD-Veh, due to levels below the detection range), Figure 4b and 4c (SCFAs and BCAAs –1 data point from HFHSD-*P. fae* due to levels below the detection range). Data shown as mean ± SEM. One-way ANOVA followed by Tukey’s *post-hoc* test (a−c); Student’s t-test (d) Significant effects at *p < 0.05, **p < 0.01 and ***p < 0.001 and ^#^*p* < 0.05, ^##^*p* < 0.01 and ^###^*p* < 0.001 versus CD-Veh group in (a−c); ^#^*p* < 0.05 versus control (PBS) in (d). See also Figure S3.

We next analyzed cecal contents for PYY secretagogues such as BAs, SCFAs and their branched forms (BCFAs), and BCAAs, associated with protein-induced anorexigenic hormone secretion^58^^,^^59^. Among all BAs analyzed (Figure 4a and S3a), we found that the concentrations of the secondary BA ωMCA and its taurine-conjugated form TωMCA, as well as the primary BAs αMCA and βMCA, were significantly higher in the cecum of untreated HFHSD-fed mice than in CD-fed mice (Figure 4a, ωMCA *p* = 0.017; TωMCA *p* = 0.006; αMCA *p* = 0.010; βMCA *p* = 0.029). Notably, *P. faecium* administration completely restored the levels of TωMCA and ωMCA to those observed in CD-fed mice (HFHSD-Veh vs HFHSD-*P. fae*, *p* = 0.013 for TωMCA and *p* = 0.048 for ωMCA), whereas the levels of αMCA and βMCA were only partially normalized and were not significantly different to those in untreated HFHSD-fed mice (Figure 4a).

Analysis of SCFAs (Figure 4b and S3b) revealed that the level of the BCFA isovalerate was significantly higher in *P. faecium*-treated HFHSD-fed mice than in untreated mice fed either HFHSD or CD (Figure 4b: vs CD-Veh, *p* < 0.001 and vs HFHSD-Veh, *p* = 0.002). Similarly, acetate levels were higher in *P. faecium*-treated mice than in CD-fed mice (Figure 4b: vs CD-Veh, *p* = 0.003), but this difference was statistically significant only when the HFHSD-fed groups were compared (Student’s t-test, *p* = 0.040) (Figure 4b). By contrast, heptanoate levels were significantly lower in untreated HFHSD-fed mice than in CD-fed mice (Figure 4b, *p* = 0.045), but this decrease was not observed in mice treated with the bacterium (Figure 4b). Assessment of BCAAs demonstrated that *P. faecium*-treated mice had higher cecal concentrations of valine, leucine and isoleucine than untreated mice fed either HFHSD or CD (Figure 4c).

To explore whether *P. faecium* affects PYY secretion via metabolites, we conducted *in vitro* experiments using the murine EEC line STC-1. For these experiments, we selected BCAAs as the most probable candidates involved, because their levels were increased by *P. faecium* administration. We also hypothesized that, in *P. faecium*-treated mice, BAs changes were less relevant to PYY secretion, as the bacterium reduced, rather than increased, their levels. These levels were enhanced in untreated HFHSD-fed mice and their reduction induced by *P. faecium* treatment was probably a consequence of the decreased lipid intake attributed to the bacterium.

Incubating the murine EEC line STC-1 with the BCAAs valine, leucine, or isoleucine increased PYY secretion over control conditions (Figure 4d: Control vs valine, *p* = 0.017; vs leucine, *p* = 0.002; vs isoleucine *p* = 0.005; and Figure S3c). Additionally, *P. faecium* itself enhanced PYY release by STC-1 cells compared with the control (Figure 4d, *p* = 0.051).

**Figure 5.**
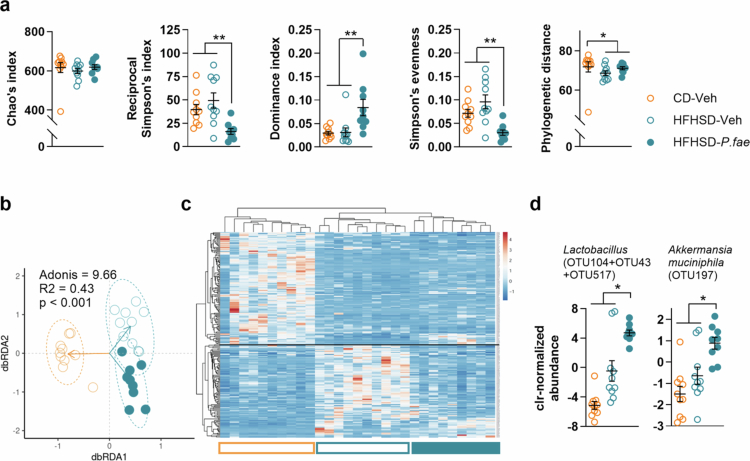
*P. faecium* drives changes in gut microbiota composition. In mice fed CD or HFHSD treated with Veh or with *P. fae*, we assessed in feces: (a) Alpha diversity, including the observed OTUs, Simpson’s reciprocal index, Simpson’s evenness index, dominance index, and phylogenetic distance descriptors. (b) Beta diversity evaluation of microbial community structure using distance-based redundancy analysis (dbRDA). The two gradients of dataset dispersion in ordination space explaining more variation are shown in a scatter-plot fashion. (c) Scaled read counts for top differentially abundant OTUs (*n* = 209, chi-squared test ≥ 11.5, corrected p ≤ 0.01) across groups are shown as a heatmap. Clustering of OTUs was carried out using Euclidean distance and the “complete” clustering method. OTUs from major clusters were identified using SINA aligner. Heat scale is based on Z-scores resulting from rarefied read counts per OTU (raw scaling). A horizontal black line divides OTUs clustering into two major groups supporting the effect of HFHSD (top half) and of *P. faecium* (bottom half). (d) Distribution of clr-normalized DNA read counts for OTUs identified as *Lactobacillus* (OTU104, OTU43, OTU517) and *Akkermanisa muciniphila* (OTU197), *n* = 9−10 per group. Kruskal-Wallis (KW) test and pairwise comparisons with respective adjusted (FDR) *p*-values are indicated in each graph (a); Adonis test with centroids of data dispersion indicated with arrow heads (b); pairwise comparisons between groups and adjusted (FDR) *p*-values (d). CD/HFHSD-Veh *n* = 10 per group and HFHSD-*P. fae* *n* = 9 (1 data point was excluded due to invalid sample collection). Abbreviations: CD: control diet; clr: centered log-ratio; HFHSD: high-fat, high-sugar diet; *P. fae*: *Phascolarctobacterium faecium*; Veh: vehicle. See also Figure S4.

To understand how *P. faecium* treatment affected the gut microbiota, we analyzed alpha and beta diversity based on the 696 different operational taxonomic units (OTUs) identified. With regards to alpha diversity, we found no significant difference in richness between the study groups, as indicated by the Chao index (KW = 4.09, *p* < 0.390), which suggests no loss of bacterial species due to the treatments (Figure 5a). However, other alpha diversity measures including the reciprocal Simpson’s index, dominance and evenness, were significantly altered by *P. faecium* administration, suggesting shifts in the relative abundance of specific OTUs (the balance among species), partially counteracting the effects observed in HFHSD-fed mice and reaching values beyond those of the CD group (Figure 5a). With regards to beta diversity, multivariate analysis using the Bray-Curtis dissimilarity index and redundancy analysis (RDA) revealed a substantial shift in the microbial community structure due to the HFHSD feeding (Adonis = 9.66, *p* < 0.001) (Figure 5b). *P. faecium* did not reverse the HFHSD-induced gut microbiota changes back to the CD state, but instead generated a distinct microbiota structure (Figure 5b). When examining the effects on individual taxa, we found that approximately one-third of the OTUs detected showed differential abundance across all experimental groups (*N* = 209) (Supplementary_Data_1.xlsx). HFHSD-feeding led to a decrease in several OTUs belonging to the Muribaculaceae family, which are predominant in murids (top-half of heatmap in Figure 5c). This loss of commensal gut bacteria was not completely restored by *P. faecium* administration; however, *P. faecium* administration did reduce the abundance of Lachnospiraceae-enriched OTUs (also including some species of the families Ruminococcaceae, Oscillospiraceae, Eggerthellaceae and Erysipelotrichaceae) that were increased in untreated HFHSD-fed mice (bottom-half of heatmap in Figure 5c), bringing their levels closer to those observed in CD-fed mice. Compared with untreated CD- and HFHSD-fed mice, *P. faecium*-HFHSD-treated mice showed a significant increase in *Lactobacillus* spp. (OTU43, OTU104 OTU517, *p* = 0.031) and *Akkermansia muciniphila* (OTU197, *p* = 0.017) (Figure 5d). Both *Lactobacillus* and *Akkermansia* species showed slight, but significant positive correlations with cecal BCAA levels: specifically, OTU104, OTU517 and OTU197 with valine (Figure S4a), leucine (Figure S4b) and isoleucine (Figure S4c). Additionally, undefined species from the family Erysipelotrichaceae, such as OTU3, OTU94 and OTU595, were significantly increased by *P. faecium* administration (KW > 15.5, *p* < 0.005), and exhibited positive correlations with BCAA cecal levels (Kendall’s tau > 0.45, *p* < 0.001) (data not shown). In another study, *P. faecium* was detected in the cecal content of *P. faecium*-treated DIO mice, whereas it remained undetectable in vehicle-treated controls (data not shown).

Altogether, these findings suggest that *P. faecium* supplementation promotes PYY secretion through several mechanisms: these likely include direct pathways involving *P. faecium* structural components and indirect pathways driven by the bacterium-induced gut microbiota and metabolite changes influencing BCAAs signaling.

### The anorexigenic effects of P. faecium help prevent diet-induced obesity in mice

To assess how the anorexigenic effects of *P. faecium* contribute to the long-term attenuation of body weight gain and adiposity in DIO (Figure 1), we conducted a controlled feeding experiment in HFHSD-fed mice using untreated and *P. faecium*-treated *ad libitum*-fed groups and an untreated pair-fed group whose food intake was restricted to match the amount consumed by *P. faecium*-treated mice.

**Figure 6.**
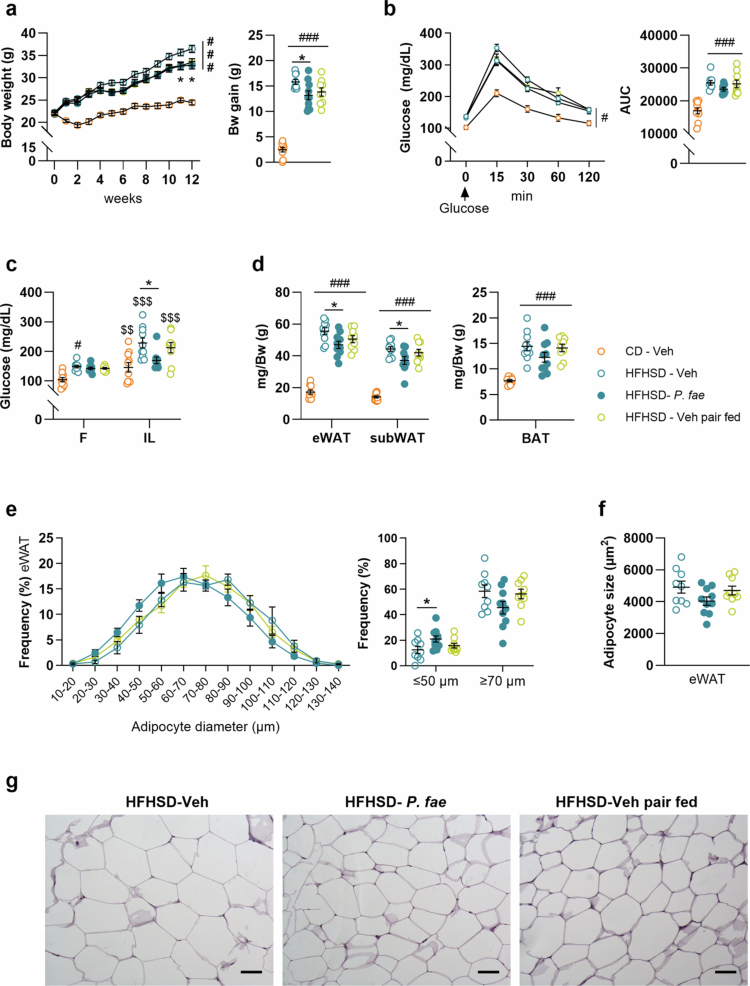
The effect of *P. faecium* reducing food intake partially prevents body weight gain, postprandial hyperglycemia and adiposity in diet-induced obese mice. In mice fed CD or HFHSD treated with Veh (fed *ad-libitum* or pair-fed as *P. fae*-treated group) or treated with *P. fae*, we measured: (a) Bw follow-up and Bw gain. (b) glycemia after 0, 15, 30, 60, and 120 min of the OGTT (2 g/kg) after 4 h of fasting, and AUC at week 10 of the intervention. (c) Blood glucose levels in fasting and 20 min after an oral load of Intralipid 20% at week 10 of the intervention. (d) Weights of BAT, eWAT and subWAT normalized by body weight, after 12 weeks of intervention. (e) Frequency of adipocytes (diameters) in eWAT after 12 weeks of intervention in HFHSD-fed mice. (f) Quantification of individual adipocyte area in eWAT and (g) representative images of hematoxylin and eosin staining (scale bar = 50 μm, 16 × magnification). Abbreviations: CD: control diet, HFHSD: high-fat, high-sugar diet; Veh: vehicle; *P. fae*: *Phascolarctobacterium faecium*; Bw: Body weight; OGTT: oral glucose tolerance test; AUC: area under the curve; BAT: brown adipose tissue; eWAT: epididymal white adipose tissue; subWAT: subcutaneous white adipose tissue; F: fasting; IL: 20 min after intralipid. CD-Veh *n* = 10, HFHSD-Veh/*P. fae* *n* = 7−10 per group and HFHSD-Veh pair fed *n* = 9. Measurements were excluded due to invalid values in Figure 6a (Bw—1 data point excluded from HFHSD-Veh and HFHSD-VEH pair fed, due to stress signs), Figure 6b (OGTT—1 data point excluded from HFHSD-Veh and HFHSD-Veh pair fed and 3 data points from HFHSD-*P. fae* due to invalid glucose administration), Figure 6c (Glycemia - 1 data point excluded from HFHSD-*P. fae* and HFHSD-Veh pair fed due to invalid intralipid administration), and Figure 6d (adiposity - 1 data point excluded from CD-Veh and HFHSD-Veh pair fed due to invalid sample collection). Data shown as mean ± SEM. One-way ANOVA followed by Tukey’s *post-hoc* test (a [Bw gain], b [AUC], d, e [right graph], f); two-way ANOVA followed by Tukey’s *post-hoc* test (a [Bw follow-up] b, c and e). Significant effects at ^$$^*p* < 0.01 and ^$$$^*p* < 0.001 versus fasting conditions; ^###^*p* < 0.001 versus CD-Veh group; and *p < 0.05. See also Figure S5a−f.

After 12 weeks of intervention, *P. faecium* treatment, but not pair-feeding, resulted in significantly decreased body weight gain when compared with untreated *ad libitum*-fed mice (Figure 6a and Table S2: HFHSD-Veh vs HFHSD-*P. fae*, *p* = 0.042 and HFHSD-Veh vs HFHSD-Veh pair fed, *p* = 0.198). We next questioned whether *P. faecium* administration influenced postprandial glucose homeostasis. Mice fed HFHSD showed higher glycemia and AUC during the OGTT compared to CD-fed mice, and neither the *P. faecium-*treated nor the pair-fed groups improved the OGTT (Figure 6b). *Post-hoc* analysis of the AUC neither revealed significant differences induced by *P. faecium* or pair-feeding compared to untreated HFHFSD *ad libitum*-fed mice. However, when analyzing only untreated (*ad libitum*-fed) and *P. faecium*-treated mice, *P. faecium* administration tended to decrease the AUC of the OGTT (Student’s t-test, *p* = 0.050), a result not seen when comparing *ad libitum* and pair-fed untreated groups (Student’s t-test, *p* = 0.810).

No differences in insulin levels were observed in fasting conditions or after 15 min of oral glucose administration across the three experimental groups fed HFHSD (Figure S5a). Likewise, glycemia was reduced 15 min after intraperitoneal insulin injection in all groups (Figure S5b). We also examined postprandial glycemia after oral administration of the 20% intralipid, a major component of the HFHSD. An interaction trend was observed between the prandial condition (fasting or intralipid) and the treatment groups (Figure 6c, *p* = 0.050). *Post-hoc* analysis revealed that, compared with fasting levels, glycemia increased 20 min after the oral intralipid load in untreated mice (CD-Veh, *p* = 0.006 and HFHSD-Veh, *p* < 0.001 and pair-fed, *p* < 0.001) but not in *P. faecium*-treated mice (Figure 6c). Additionally, fasting blood glucose levels were slightly reduced in *P. faecium*-treated and pair-fed groups, as these levels were only higher in HFHSD *ad libitum*-fed mice compared to CD-fed mice (Figure 6c, *p* = 0.035). Notably, after intralipid administration glycemia was lower in *P. faecium*-treated mice than in untreated mice fed a HFHSD *ad libitum* (Figure 6c, *p* = 0.004).

Analysis of WAT components revealed that the weight of eWAT and subWAT was significantly lower in *P. faecium*-treated mice than in untreated HFHSD *ad libitum*-fed mice, but not in pair-fed mice [Figure 6d and Table S2 (eWAT: HFHSD-Veh vs HFHSD-*P. fae*, *p* = 0.018 and HFHSD-Veh vs HFHSD-Veh pair fed, *p* = 0.301; subWAT: HFHSD-Veh vs HFHSD-*P. fae*, *p* = 0.031; HFHSD-Veh vs HFHSD-Veh pair fed, *p* = 0.235). The weight of BAT was not affected by either *P. faecium* treatment or pair-feeding (Figure 6d and Table S2). Analysis of adipocytes in hematoxylin-eosin stained eWAT, in HFHSD-fed groups, suggested a potential interaction between treatment groups and adipocyte size frequency (*p* = 0.080) (Figure 6e). Specifically, *P. faecium* treatment, but not pair-feeding, increased the frequency of smaller adipocytes (≤50 µm) compared with untreated *ad libitum*-fed mice (Figure 6e and 6g, *p* = 0.044). Furthermore, *P. faecium*-treated mice tended to have smaller adipocytes in eWAT than untreated *ad libitum*-fed mice (*p* = 0.090, Figure 6f), while this was not observed in pair-fed mice. In subWAT, neither the distribution of adipocyte sizes nor the average adipocyte size itself was significantly altered in either treatment group (Figure S5c-S5e). Gene expression analysis of molecular markers in subWAT revealed increased *β*-oxidation and reduced lipogenesis and thermogenesis in all HFHSD-fed groups compared to CD-fed mice, with no specific changes attributable to *P. faecium* or pair feeding (Figure S5f).

These findings again demonstrated that *P. faecium*-treated mice showed improved metabolic phenotype compared to untreated HFHSD *ad libitum*-fed mice. Nevertheless, pair-fed mice did not show significant differences compared to both untreated and *P. faecium*-treated mice fed *ad libitum* in the explored metabolic parameters. This suggests that the anorexigenic effect of *P. faecium* contributes to improved energy metabolism but is not the sole mechanism, as pair-fed mice showed an intermediate phenotype.

### P. faecium accelerates gastrointestinal transit in diet-induced obese mice and reduces lipid absorption in colonocytes

Because the reduction in food intake alone did not fully explain how *P. faecium* prevented increased body weight and adiposity in response to HFHSD-feeding, we investigated other pathways involved in energy homeostasis, including thermogenesis and intestinal functions, such as gastrointestinal transit and lipid absorption.

**Figure 7.**
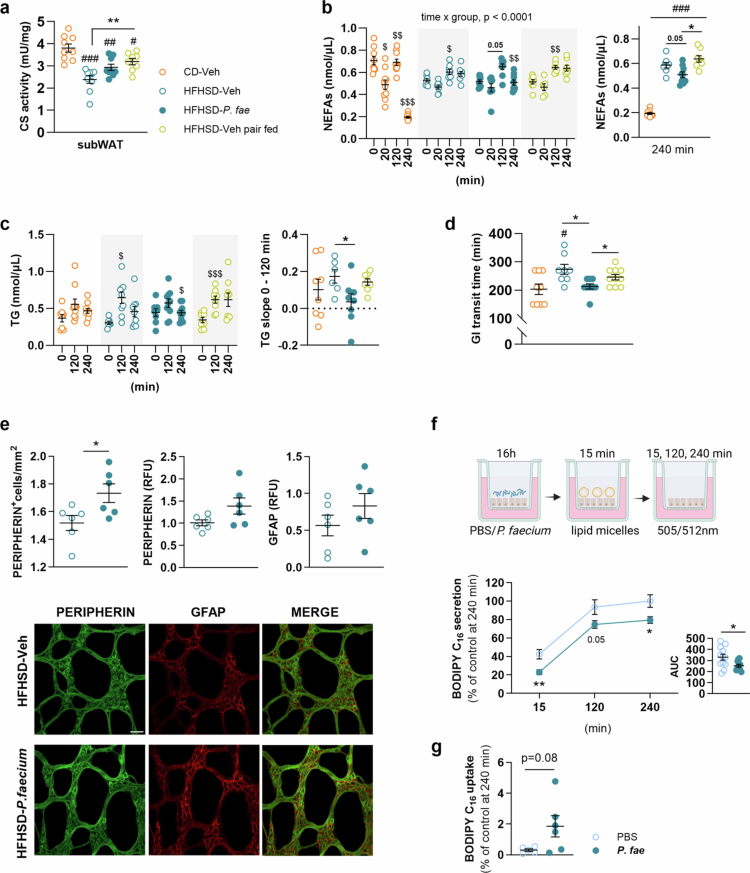
*P. faecium* accelerates clearance of non-esterified fatty acids in blood and gastrointestinal transit, and limits intestinal lipid absorption *in vitro.* In mice fed HFHSD treated with Veh (fed *ad-libitum* or pair-fed as *P. fae*-treated group) or treated with *P. fae*, we measured: (a) Citrate synthase activity in subWAT. (b) Plasma NEFAs and (c) TGs after an oral load of intralipid 20%. (d) Gastrointestinal transit time: time between oral gavage and first appearance of dye in feces in mice fed *ad libitum.* (e) Number of peripherin-positive neurons and protein expression of peripherin and GFAP in colonic CMMP whole-mount preparations (f) BODIPY C_16_ secreted into the basolateral compartment by Caco-2 cells at 4 h after addition of lipid micelles, and AUC. (g) BODIPY C_16_ uptake by Caco-2 cells at 240 min after addition of lipid micelles. Amount of BODIPY C_16_ is expressed as percentage (%) fluorescence relative to control (PBS). Abbreviations: HFHSD: high-fat, high-sugar diet; Veh: vehicle; CS: citrate synthase; CMMP: circular muscle myenteric plexus; NEFAs: non-esterified fatty acids; *P. fae*: *Phascolarctobacterium faecium*; TG: triglycerides; AUC: area under the curve; RFU: relative fluorescence units. a-d: CD-Veh, HFHSD-*P. fae* and HFHSD-Veh pair fed *n* = 8−10 per group and HFHSD-Veh *n* = 7-10; e: *n* = 6 per group; f: PBS *n* = 12 and *P. fae* *n* = 10; **g**: PBS *n* = 5 and *P. fae* *n* = 6. Measurements were excluded due to invalid values in Figure 7a (Citrate synthase activity—1 data point excluded from CD/HFHSD-Veh and HFHSD-*P. fae* and 2 data points from HFHSD-Veh pair fed due to values below the detection range), Figure 7b (NEFAs—1 data point from CD-Veh, 2 or 3, data points from HFHSD-Veh/P. fae, and 2 or 4 data points from HFHSD-Veh pair fed due to invalid blood collection), Figure 7c (TGs—2 data points from CD-Veh and HFHSD-*P. fae*, 1 or 3 data points from HFHSD-Veh and 1 or 2 data points from HFHSD-Veh pair fed, due to invalid blood collection), and Figure 7d (Gut transit—1 data point from CD/HFHSD-Veh and HFHSD-Veh pair fed, and 2 data points from HFHSD-*P*. fae due to inadequate carmine red administration). Data shown as mean ± SEM. One-way ANOVA followed by Tukey’s *post-hoc* test (a, d and right graphs in b and c); two-way ANOVA followed by Tukey’s *post-hoc* test (b, c and f); unpaired Student’s t-test (e, f [AUC] and g). Significant effects at ^$^*p* < 0.05, ^$$^*p* < 0.01 and ^$$$^*p* < 0.001 versus previous time point: ^#^*p* < 0.05, ^##^*p* < 0.01 and ^###^*p* < 0.001 versus CD-group; *p < 0.05 and ***p < 0.001. Scale bar (50μm) is represented only in the first photograph. See also Figure S5g, S5h and S6.

Gene expression analysis of thermogenesis markers in subWAT revealed no significant alterations in response to the bacterium or pair-feeding (Figure S5f). However, citrate synthase activity, a proxy for mitochondrial density frequently employed to assess thermogenesis,^60^^,^^61^ was reduced in all HFHSD-fed groups compared to CD-fed mice (Figure 7a: vs HFHSD-Veh, *p* < 0.001,; vs HFHSD-*P. fae*, *p* = 0.003; vs HFHSD-Veh pair fed, *p* = 0.049). Pair-feeding increased the activity of this enzyme compared to untreated HFHSD *ad libitum* fed mice (Figure 7a, *p* = 0.008), while a tendency to increase this activity was observed due to *P. faecium* treatment (Figure 7a, *p* = 0.090).

To investigate the role of *P. faecium* in intestinal lipid absorption and clearance, we measured plasma levels of NEFAs and triglycerides in mice fed HFHSD for 12 weeks following an intralipid oral load.

Compared with fasting levels (0 min), only CD-fed mice showed a significant reduction of circulating NEFAs 20 min after intralipid administration (Figure 7b, *p* = 0.011), indicating functional inhibition of fasting-induced lipolysis after intralipid intake in the CD group but not in any of the HFHSD-fed groups. All groups exhibited an increase in plasma NEFA levels 120 minutes after intralipid administration (Figure 7b, 20 min vs 120 min: *p* = 0.068 for CD-Veh; *p* = 0.016 for HFHSD-Veh, *p* = 0.053 for HFHSD-*P. fae*; *p* = 0.004 for HFHSD-Veh pair fed), whereas only the CD-fed and *P. faecium*-treated groups exhibited a decrease in NEFAs after 240 minutes compared with their levels after 120 minutes (Figure 7b, 120 min vs 240 min: *p* < 0.001 for CD-Veh; *p* = 0.009 for HFHSD-*P. fae*). Moreover, statistical analysis revealed an interaction between time and treatment groups (*p* < 0.001), and *post-hoc* analysis further showed that *P. faecium*-treated mice exhibited reduced NEFA levels 240 minutes after the intralipid oral load compared with untreated mice fed *ad libitum* or pair-fed mice (Figure 7b, right graph: *p* = 0.053 vs HFHSD-Veh; *p* < 0.001 vs HFHSD-Veh pair fed). These findings suggest that *P. faecium* treatment facilitates a more rapid clearance of circulating postprandial NEFAs, independent of food intake.

Plasma triglyceride levels were increased in untreated mice *ad libitum* fed HFHSD and in pair-fed mice 120 minutes after intralipid administration compared with fasting levels (Figure 7c, 0 min vs 120 min: *p* = 0.026 for HFHSD-Veh; *p* < 0.001 for HFHSD-Veh pair fed), while no changes were detected in equivalent *P. faecium*-treated and CD-fed mice. In addition, compared to untreated HFHSD *ad libitum* fed mice, the *P. faecium* group showed a reduced rate of increase for triglycerides between 0 and 120 minutes (slope) compared to untreated HFHSD *ad libitum*-fed mice (Figure 7c, right graph: *p* = 0.029), supporting a reduced absorption. NEFAs and triglyceride plasma levels in response to intralipid administration were also measured after 4 weeks of HFHSD-feeding, but no significant differences were found between groups (Figure S5g and S5h).

Given the association of obesity with prolonged gastrointestinal transit time, which can lead to increased intestinal lipid absorption and subsequent body weight gain,^62^ we assessed gastrointestinal transit time after 7 weeks of HFHSD-feeding. Only untreated mice fed HFHSD *ad libitum* showed increased gastrointestinal transit time compared to CD-fed mice (Figure 7d, *p* = 0.035). In addition, *P. faecium*-treated mice exhibited a faster transit time compared with untreated mice fed HFHSD *ad libitum* or pair-fed (Figure 7d: HFHSD-Veh vs HFHSD-*P. fae*, *p* = 0.033; HFHSD-*P. fae* vs HFHSD-Veh pair fed, *p* = 0.027). This indicated that, independent of food intake, the bacterium accelerates gut transit, potentially influencing intestinal lipid absorption. Immunofluorescent analysis of the neuronal marker peripherin in colonic myenteric plexus, in untreated and *P. faecium*-treated HFHSD *ad libitum* fed mice, revealed that the bacterium increased the number of peripherin-positive cells (Figure 7e, *p* = 0.033) and tended to enhance peripherin expression (Figure 7e, *p* = 0.070). No significant changes were observed in the expression of the glial marker GFAP (Figure 7e). To note, we also found that, compared to CD, *ad libitum* HFHSD feeding led to a reduction in peripherin protein expression (Figure S6).

To investigate the impact of *P. faecium* on lipid absorption in the distal gut, we used Caco-2 cells cultured in Transwell plates and incubated with fluorescent fatty acid BODIPY™ FL C_16_. Compared with the control, pre-treatment with *P. faecium* (16 hours) resulted in significantly decreased secretion of BODIPY™ FL C_16_ into the basolateral compartment (Figure 7f: 15 min, *p* = 0.004; 120 min: *p* = 0.051; 240 min: *p* = 0.018), while the amount of non-absorbed BODIPY™ FL C_16_ in the apical compartment remained unaffected by the bacterium (not shown). BODIPY™ FL C_16_ cellular uptake showed a non-significant trend for increased uptake at 240 min in *P. faecium*-treated Caco-2 cells compared with the control (Figure 7g, Student’s t-test, *p* = 0.080).

Overall, our data suggest potential mechanisms by which *P. faecium* may attenuate DIO independently of its effects on food intake. These mechanisms include accelerated gastrointestinal transit —likely mediated through protection of the enteric nervous system— along with improved lipid clearance, which could contribute to limiting fat accumulation.

## Discussion

This study reveals how commensal gut bacteria may protect against obesity by interacting with the enteroendocrine system and intestinal transit. Specifically, *P. faecium* provides metabolic benefits by suppressing appetite *via* increased PYY secretion in early-stage DIO in mice. Furthermore, *P. faecium* influences intestinal functions that help alleviate obesity, including accelerating intestinal transit and limiting intestinal lipid absorption, leading to enhanced postprandial lipid clearance.

Our study provides strong evidence that *P. faecium* has appetite-suppressing effects in DIO mice, highlighting its potential for obesity management. We demonstrate that *P. faecium* prevents hyperphagia in DIO mice, associated with increased plasma levels of the anorexigenic hormone PYY and a greater number of PYY-producing cells in the colon. These findings align with other research supporting the role of gut bacteria in obesity treatment; for example, preclinical and clinical studies have shown that the commensal intestinal bacterium *Hafnia alvei* 4597 can aid in weight management due to the anorexigenic effects of its heat shock protein ClpB, an antigen-mimetic of *α*-MSH.^63^^,^^64^Other studies have identified intestinal bacteria with PYY secretagogue capacity.^65-67^ For instance, Liang et al. reported that oral supplementation with *Ligilactobacillus salivarius* LCK11 increased intestinal and plasma PYY levels in mice after 8 weeks.^67^ However, the direct causal link between that bacterium and obesity has yet to be definitively established using loss-of-function models.

Oral administration of *P. faecium* increased the plasma levels of PYY but not GLP-1. The elevated plasma levels of PYY, observed from the early stages of obesity development through the end of the intervention, strongly suggest a primary role of PYY in the ability of *P. faecium* to modulate food intake. Consistent with preventing hyperphagia, *P. faecium* appeared to restore hypothalamic control of food intake. This was evidenced by the fact that *P. faecium*-treated mice did not overexpress the anorexigenic neuropeptide POMC, a compensatory mechanism seen in untreated obese mice in response to excessive energy intake. Our findings indicate that early hypersecretion of PYY explains the anorexigenic effects of the bacterium, as immunoneutralization of PYY after 4 weeks of treatment blunted the food intake-suppressing effect of the bacterium. Furthermore, *P. faecium* only suppressed food intake during the dark phase, aligning with our observation that PYY secretion in response to lipid intake occurs specifically during this period. *P. faecium* was administered immediately prior to the onset of the active phase, suggesting that its effect on reducing food intake may be transient. Nevertheless, further studies are warranted to determine whether the anorexigenic properties of this bacterium depend on the timing of administration.

Additionally, the finding that the anorexigenic effects of *P. faecium* occur during the active period in rodents suggests that it may require active food consumption. This could enhance the intestinal availability of dietary and/or microbial molecules to activate gut-to-brain nutrient sensing, in line with the bacterial growth dynamic-based model of appetite control.^35^^,^^68^ Although we did not specifically explore light/dark variations in intestinal metabolites, we did find increased levels of the BCAAs valine, leucine, and isoleucine in the cecum of *P. faecium*-treated mice. This increase could stem from the ability of *P. faecium* to shape the gut microbiota by decreasing the population of bacterial species involved in BCAA uptake and degradation,^69-71^ while simultaneously increasing those involved in *de novo* BCAA biosynthesis.^69^^,^^70^ Indeed, we found a higher abundance of *Akkermansia muciniphila* in *P. faecium*-treated mice, a bacterium known to synthesize BCAAs from glucose,^72^ and which showed a positive correlation with cecal BCAAs levels. While BCAAs have been associated with insulin resistance, glucose intolerance and obesity,^70^^,^^73-78^ other studies have demonstrated metabolic benefits. For instance, isoleucine reduced postprandial glycemia in rats,^79^^,^^80^ and leucine and isoleucine supplementation reduced body weight gain and fat mass in DIO mice.^81-83^ Our mechanistic studies using STC-1 neuroendocrine cells suggest that the increase in PYY release may be mediated by a BCAA-dependent pathway, as these metabolites can act as PYY secretagogues. In line with our findings, Genton et al. reported that 4-month oral supplementation with valine, leucine and isoleucine increased plasma PYY levels in patients with chronic kidney disease,^59^ a frequent complication of type 2 diabetes.^84^

*P. faecium* could also be the direct mediator of the PYY increase, as we found that it stimulates PYY secretion by SCT-1 cells. Similar *in vitro* studies have demonstrated the PYY secretagogue properties of *Ligilactobacillus salivarius* LCK11, mediated by the ability of its peptidoglycan to activate TLR2/NF-κB signaling.^67^ Given the expression of TLRs in EECs^32^^,^^33^^,^^67^ and their role in promoting *Pyy* gene expression in L cells,^85^ TLR ligands could mediate the effects of *P. faecium* on the enteroendocrine system to boost PYY release. Notably, *P. faecium* prevented exacerbated energy intake from the early stages of HFHSD feeding, indicating that modulating feeding behavior is a key mechanism through which the bacterium prevents metabolic alterations. Indeed, body weight changes and plasma levels of triglycerides and cholesterol improved after only 4 weeks of treatment.

We investigated whether the ability of the bacterium to regulate food intake contributes to obesity prevention by conducting a pair-feeding experiment. Pair-fed and *P. faecium*-treated mice showed similar decreases in body weight, adiposity and postprandial glycemia. These similarities are particularly evident for body weight, suggesting that the bacterium ´ s effect in attenuating body weight gain in DIO is influenced, at least in part, by food intake. The limitations inherent of the pair-feeding paradigm, including altered feeding behavior and hormonal secretion caused by food restriction, might mask the precise contribution of food intake to the effects of *P. faecium* on energy homeostasis.

Pair-feeding did not fully replicate the improved metabolic phenotype induced by *P. faecium*. In contrast to *P. faecium* treatment, there were no significant differences between pair-fed and untreated *ad libitum*-fed mice in body weight gain, fat depot weight, number of small adipocytes in eWAT, and glycemia in response to lipid intake. Therefore, we hypothesize that *P. faecium* also triggers food intake-independent mechanisms that collectively contribute to its metabolic benefits.

We next investigated additional mechanisms that could mediate the observed metabolic improvement, specifically examining effects on gastrointestinal transit and lipid absorption. Gastrointestinal motility is often impaired in obesity, characterized by delayed transit and reduced peristalsis due to the reduction of enteric neurons induced by hypercaloric diets, ^86^^,^^87^ leading to a longer timeframe for nutrient absorption and body weight gain.^62^^,^^88-90^ The pair-feeding protocol indicated that, independent of lower food intake, *P. faecium* accelerated gastrointestinal transit, probably due to its effect increasing the enteric neurons density in the circular muscle myenteric plexus in colon. This may accelerate the movement of nutrients through the intestine, resulting in a greater quantity of unabsorbed dietary products reaching the colon. This could represent a source of BCAAs that, together with BCAAs synthesized *de novo* by gut bacteria, could contribute to a greater BCAA pool to subsequently stimulate PYY secretion.

Irrespective of food intake, the ability of *P. faecium* to accelerate gastrointestinal transit could limit intestinal lipid absorption, thereby helping to prevent increased fat storage. Consistent with this, both 4 and 12 weeks of *P. faecium* supplementation reduced the weight of subWAT. The 12-week supplementation also resulted in lower eWAT weight and a higher frequency of smaller adipocytes. Furthermore, *P. faecium* prevented, albeit modestly, the 120 minutes postprandial triglyceride peak seen in untreated DIO mice after intralipid administration, suggesting lower intestinal lipid absorption. *P. faecium* also induced earlier clearance of postprandial NEFAs from blood 240 minutes after intralipid administration, indicating faster NEFA storage in adipose tissue. Considering the lower fat depot weights in *P. faecium*-treated mice, this quicker NEFA clearance appears to prevent the HFHSD-induced dyslipidemia without contributing to adiposity.

Assuming that accelerated gastrointestinal transit could increase the amount of unabsorbed lipids reaching the colon in mice treated with *P. faecium*, we investigated its effects on lipid absorption in the distal gut. Our *in vitro* experiments demonstrated that *P. faecium* enhanced cellular lipid uptake and utilization by colonocytes. Specifically, the bacterium prevented the crossing of lipids from the apical to basolateral sides of Caco-2 cells, thereby limiting lipid absorption and helping to prevent increased adiposity. Interestingly, some gut bacteria have been shown to promote lipid storage in enterocytes, restricting chylomicron secretion.^91^

The mechanism by which *P. faecium* modulates intestinal physiology to control energy homeostasis independently of food intake warrants further investigation. However, in light of our results, we can speculate that these effects can be partially driven by *P. faecium*-induced changes in the gut microbiota. These could reinforce the beneficial metabolic effects of *P. faecium* in DIO mice, thereby explaining part of the metabolic discrepancies between pair-fed mice and *P. faecium*-treated mice. Interactions through cross-feeding mechanisms between *A. muciniphila* and *P. faecium* are plausible since *A. muciniphila* is a succinate producer and *P. faecium* is a succinate consumer.^92^ Additionally, *A. muciniphila* grown in a mucin-supplemented medium has been shown to increase the production of leucine and valine,^93^ which could explain the observed increase in BCAAs in our study. These mechanisms could facilitate metabolic cooperation between *P. faecium* and *A. muciniphila*, ultimately influencing host energy homeostasis in *P. faecium*-treated mice. Additionally, *P. faecium* may directly impact gut physiology through either intestinal colonization or transient passage, as its presence was confirmed in the cecal content of treated DIO mice (unpublished data).

Overall, our preclinical findings suggest that the anorexigenic effects of *P. faecium*, mediated by PYY hypersecretion, contribute to improved body weight control in diet-induced obese mice. The ability of the bacterium to also accelerate gastrointestinal transit time and reduce fat absorption further supports its contribution to a healthier metabolic profile. Consequently, our findings could potentially inform the future development of new therapies aimed at modulating food intake and eating behavior (major contributors to obesity), as well as intestinal transit and fat absorption. However, the translational potential should be interpreted cautiously, considering the inherent limitations of the present study model, which does not replicate the complexity of the human gut microbiota and human physiology, and the need to establishing clinically relevant dose equivalence, and assessing possible colonization or persistency of the bacterium in the intestine.

## Supplementary Material

Supplementary material Supplementary_Data_1.

Supplementary material Supplementary_Data_2.

Supplementary material Supplemental_material_clean.docx

## Data Availability

Data are available as Supplementary_Data_1.xlsx and Supplementary_Data_2.xlsx. The raw sequencing data derived from microbiota analysis on mice cecal content is publicly available at the European Nucleotide Archive (ENA) via accessing the bioproject PRJEB66189.

## Abbreviations

BAs: bile acids
BAT: brown adipose tissue
BCAAs: branched amino acids
BCAAs: branched-chain amino acids
CD: control diet
CMMP: circular muscle myenteric plexus
DIO: diet-induced obese
EECs: enteroendocrine cells
eWAT: Epididymal white adipose tissue
GLP-1: glucagon-like peptide 1
GPCRs: G-protein coupled receptors
HFHSD: high-fat high-sugar diet
NEFAs: non-esterified fatty acids
OGTT: oral glucose tolerance test
OTU: Operational Taxonomic Unit
PYY: peptide YY
SCFAs: short-chain fatty acids
subWAT: subcutaneous white adipose tissue
TLRs: toll-like receptors
ZT: zeitgeber time
