# Supplementary material for: Phascolarctobacterium faecium reduces food intake via PYY signaling, contributing to the mitigation of body weight gain in diet-induced obese mice
Source: Gut Microbes. 2026 Jan 21;18(1):2617691. doi: 10.1080/19490976.2026.2617691 (PMC12834159; doi:10.1080/19490976.2026.2617691)
Supplement: Supplementary material — Supplemental_material_clean.docx [file KGMI_A_2617691_SM1708.docx]

**Supplemental information**

**Figure S1. Graphical representation of the experimental design**

**(a)** Experiment 1 (effects of *P. faecium* in diet-induced obese mice): 4-, 8- and 12-week interventions with oral supplementation of *P. faecium* to diet-induced obese mice. Body weight data for the 12-week intervention were obtained from a separate cohort (CD/HFHSD-Veh n=10 per group and HFHSD-*P. fae* n=7). **(b)** Experiment 2 (pair-feeding experiment): 12-week intervention with oral supplementation of *P. faecium* to diet-induced obese mice, including a group pair-fed with the *P. faecium*-treated group. **(c)** Experiment 3 (PYY-blocking experiments in HFHSD-fed mice). Validation of PYY immunoneutralization with a PYY-antibody (1^st^: PYY-antibody dose-testing experiment; 2^nd^: PYY-antibody efficacy testing experiment with exogenous PYY) and PYY-blocking experiment in *P. faecium*-treated mice. Total number of mice: 162. Potential confounders, including the order of treatments and timing of measurements were minimized through randomization and a counterbalanced design. The researchers were not blinded to group allocation. All the animals were included in the analysis, but for the biochemical, hormonal and molecular measurements, data below the limit of detection were excluded. Abbreviations: Bw: body weight; CD-Veh: control diet-vehicle, CMMP: circular muscle myenteric plexus**,** HFHSD-Veh: high-fat, high-sugar diet-vehicle, HFHSD-*P. fae*: high-fat, high-sugar diet-*P. faecium*; ip: intraperitoneal.

**Figure S2. PYY secretion in response to intralipid, and efficacy of PYY immunoneutralization on food intake**

**(a)** Plasma levels of PYY at fasting and 20 min after an oral load of intralipid 20% at the onset of the light and dark phase in 4-h-fasted mice fed HFHSD for 4 weeks (n=7-8). **(b)** 12-h food intake of HFHSD-fed mice intraperitoneally injected with saline, 10× (1500 pg/mouse) or 100× (15000 pg/mouse) anti-PYY antibody 1 h before the onset of the dark phase (n=6). **(c)** 4-h-food intake of HFHSD-fed mice receiving saline or 10× anti-PYY antibody 1 h before the onset of the dark phase and injected with exogenous PYY_3-36_ (5 µg/kg body weight). Data are shown as mean ± SEM. **a:** HFHSD-Veh n=9-10, HFHSD-*P. fae* n=10; **b**: n=6 per group; **c:** n=8. Abbreviations: F: fasting; FI: food intake; IL: 20 min after oral load of intralipid; Sal: saline; w: week. Three-way ANOVA followed by Tukey´s *post-hoc* test (**a**); one-way ANOVA followed by Tukey´s *post-hoc* test (**b**); one-way repeated measures ANOVA followed by Tukey´s *post-hoc* test (**c**); significant main effects are shown in the graph. *p<0.05, **p<0.01 and ***p<0.001.


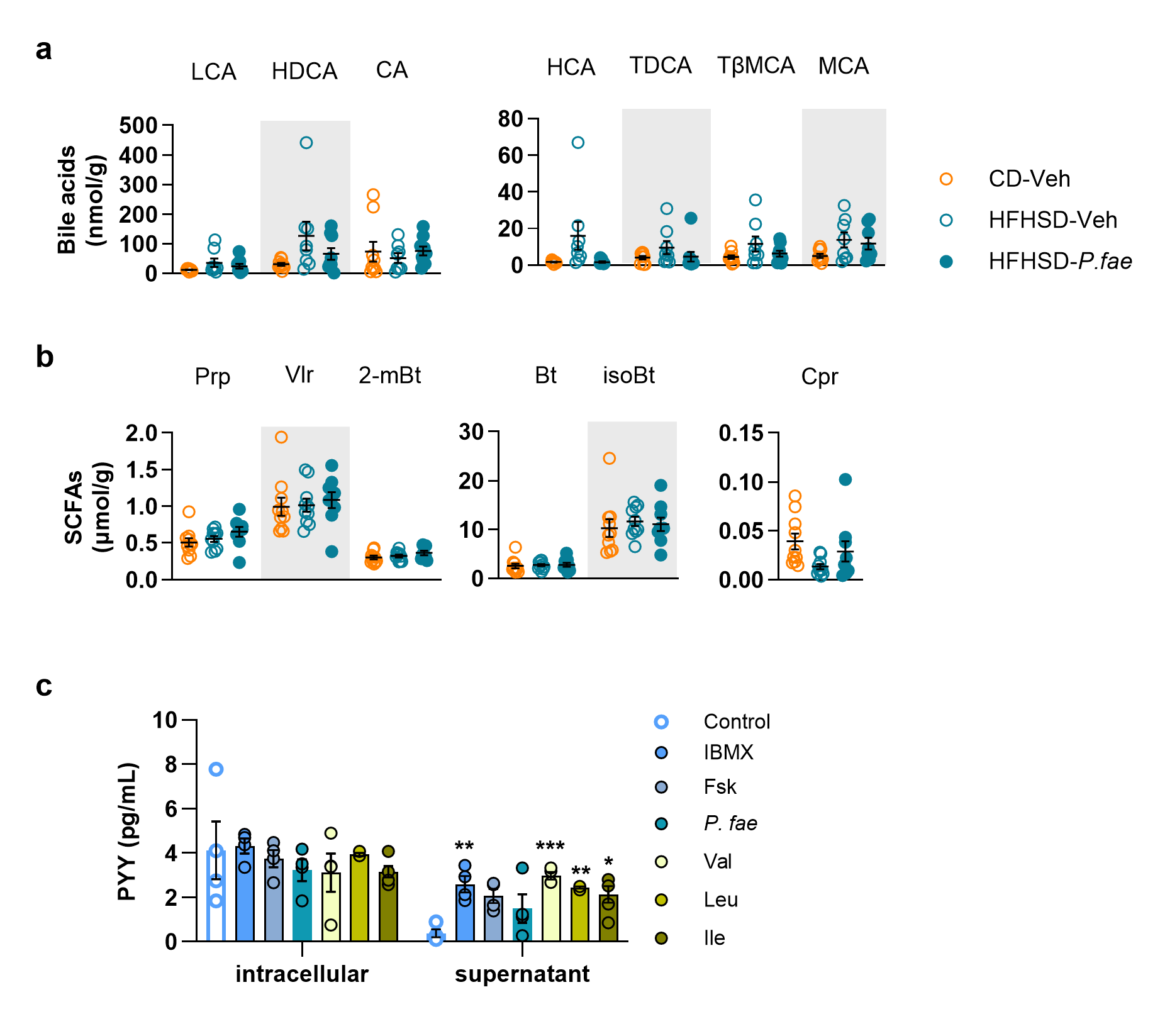


**Figure S3. Assessment of bile acid and short-chain fatty acid levels in the cecum, and PYY concentration in STC-1 cells in vitro, in response to *P. faecium* and branched-chain amino acids**

**(a)** Levels of bile acids and **(b)** short-chain fatty acids in cecum content of mice fed CD or HFHSD, treated with vehicle or *P. faecium* at 12 weeks of intervention. **(c)** PYY concentration in cell lysates (intracellular) and in supernatants after incubation with IBMX, Fsk, *P. faecium* and BCAAs. Data are shown as mean ± SEM. **a and b**: CD-Veh n=9-10, HFHSD-Veh n=8-10 and HFHSD-*P. fae* n=9; **c:** n = 4 for all conditions, except Leu (n = 3) and Ile (n = 5) (Abbreviations: 2-mBt: 2-methylbutyrate; Bt: butyrate; CA: cholic acid; CD: control diet; Cpr: caproate; Fsk: forskolin; HCA: hyocholic acid; HDCA: hyodeoxycholic acid; HFHSD: high fat high sugar diet; IBMX; 3-Isobutyl-1-Methylxanthine; Ile: isoleucine; isoBt: isobutyrate; LCA: lithocholic acid; Leu: leucine; MCA: muricholic acid; *P. fae*: *Phascolarctobacterium faecium*; Prp: propionate; TDCA: taurodeoxycholic acid; TβMCA: tauro-β-muricholic acid; Val: valine; Veh: vehicle; Vlr: valerate. One-way ANOVA followed by Tukey´s *post-hoc* test (**a** and **b**) and unpaired Student’s t-test (**c**).

**Figure S4. Correlations between the levels of branched-chain amino acids and the abundance of bacterial species increased in the cecum of *P. faecium*-treated mice [*Lactobacillus* species (OTU104, OTU43, OTU517) or *Akkermansia muciniphila* (OTU197)]**

**(a)** Correlations between the abundance of *Lactobacillus* species (OTU104, OTU43, OTU517) or *Akkermansia muciniphila* (OTU197) and Val, **(b)** Leu, and **(c)** Ile in cecum content of mice at 12 weeks of intervention. **a-c:** CD/HFHSD-Veh n=10 per group and HFHSD-*P. fae* n=9. Abbreviations: clr: centered log-ratio, Ile: isoleucine; Leu: leucine; Val: valine; τ: Kendall’s tau. Kendall rank correlations. *p<0.05 and **p<0.01.


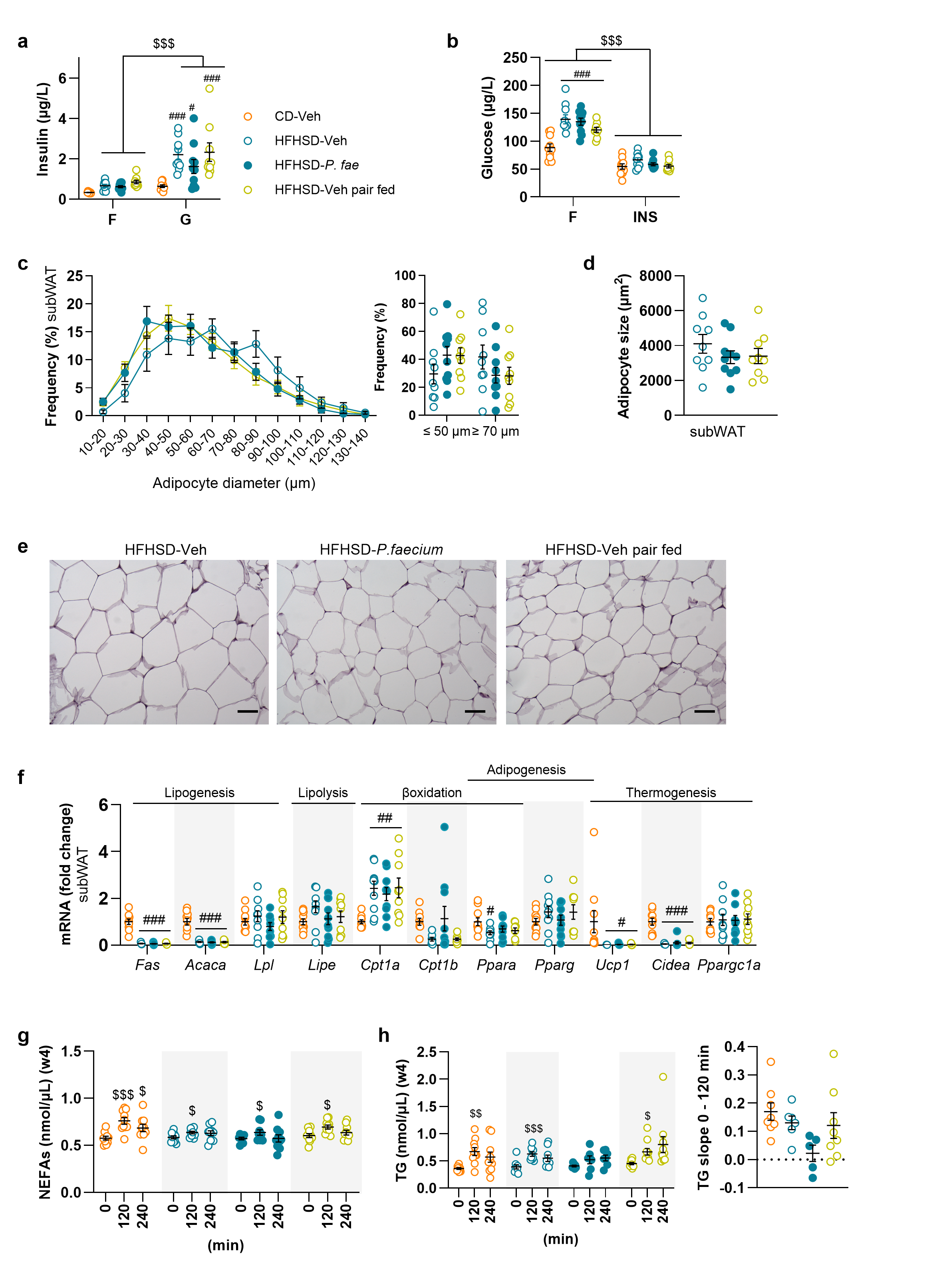


**Figure S5. Glucose clearance, adipocyte morphology, lipid metabolism-related gene expression, and circulatory lipid levels in *P. faecium*-treated and pair-fed mice**

**(a)** Plasma insulin levels in fasting and 15 min after an oral glucose load (2 g/kg). **(b)** Blood glucose levels in fasting and 15 min after an i.p. injection of insulin (1.5 U/kg). **(c)** Frequency of adipocyte sizes in subWAT**. (d)** Quantification of individual adipocyte area in subWAT and **(e)** representative bright field images of hematoxylin and eosin staining (scale bar = 50 μm, 16× magnification). **(f)** Gene expression analysis of markers of lipogenesis, lipolysis, β-oxidation, adipogenesis and thermogenesis in subWAT. **(g** and **h)** Plasma non-esterified fatty acids and triglycerides in fasting (0 minutes) and 120 and 240 minutes after an oral load of intralipid 20%. Abbreviations: HFHSD: high fat high sugar diet; Veh: vehicle; *P. fae:* *Phascolarctobacterium faecium*; F: fasting; G: 15 min after glucose load; INS: 15 min after insulin injection; NEFAs: non-esterified fatty acids; TG: triglycerides. Data are shown as mean ± SEM. CD/HFHSD-Veh and HFHSD-Veh pair fed n=9-10 per group; HFHSD-*P. fae* n=6-10. Two-way ANOVA followed by Tukey´s *post-hoc* test (**a**, **b**, **c**, **g** and **h**); One-way ANOVA followed by Tukey´s *post-hoc* test (**c:** right graph, **f**, and **h**, right graph). ^$^p<0.05 and ^$$$^p<0.001 versus previous time point or fasting, and ^#^p<0.05, ^##^p<0.01, and ^###^p<0.001 versus CD-group.

**
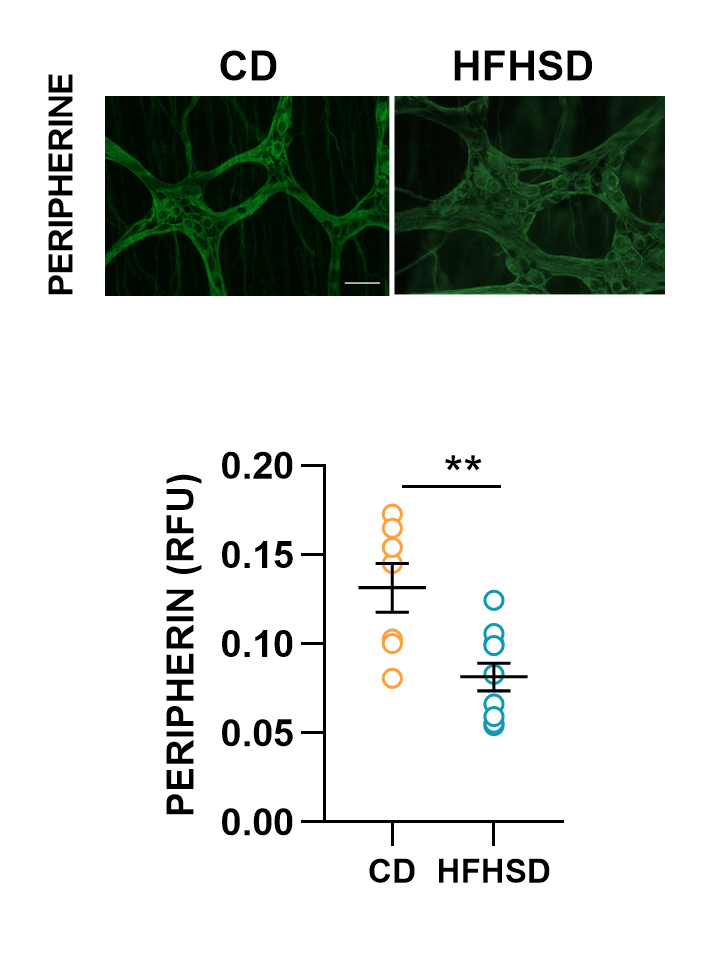
**

**Figure S6.** Protein expression of peripherin in colonic CMMP whole-mount preparations of mice fed control or high-fat high-sugar diets for 7 weeks (A separate cohort was used for this analysis). Abbreviations: CD: control diet; HFHSD: high-fat high-sugar diet; RFU: relative fluorescence units. Data shown as mean ± SEM. CD n=7 and HFHSD n=10. Unpaired Student´s t test, *p<0.05. Scale bar (50μm) is represented only in the first photograph.

**Table S1. Genes, primer pair sequences and qPCR conditions**

| **Gene name** | **Abbreviation** | **Primers sequence 5'-3'** | |
| --- | --- | --- | --- |
| **Hypothalamus** |  |  |  |
| Agouti-related neuropeptide | ***Agrp*** | Forward | AGGTCTAAGTCTGAATGGC |
|  |  | Reverse | CGGTTCTGTGGATCTAGC |
| Cocaine- and amphetamine-regulated transcript | ***Cart*** | Forward | AAGAAGTCCTGAAGAAGCTC |
|  |  | Reverse | CAAGCACTTCAAGAGGAAAG |
| Neuropeptide Y | ***Npy*** | Forward | AATCTCATCACCAGACAGAG |
|  |  | Reverse | CTTTCCTTCATTAAGAGGTCTG |
| Pro-opiomelanocortin-alpha | ***Pomc*** | Forward | AAAAGAGGTTAAGAGCAGTG |
|  |  | Reverse | ACATCTATGGAGGTCTGAAG |
| **Intestine** |  |  |  |
| Peptide YY | ***Pyy*** | Forward | CTTCACAGACGACAGCGACA |
|  |  | Reverse | GGGAAATGAACACACACAGCC |
| NeuroD1 | ***Neurod1*** | Forward | AGGAATTCGCCCACGCAGAAG |
|  |  | Reverse | CTCCTCTGCATTCATGGCTTCAAG |
| Neurogenin-3 | ***Ngn3*** | Forward | ACTCAGCAAACAGCGAAGAAG |
|  |  | Reverse | CAGTGCCCAGATGTAGTTGTG |
| **Adipose tissue** |  |  |  |
| Acetyl-Coenzyme A carboxylase alpha | ***Acaca*** | Forward | TAATGGGCTGCTTCTGTGACTC |
|  |  | Reverse | CTCAATATCGCCATCAGTCTTG |
| Cell death-inducing DNA fragmentation factor alpha-like effector A | ***Cidea*** | Forward | TGCTCTTCTGTATCGCCCAGT |
|  |  | Reverse | GCCGTGTTAAGGAATCTGCTG |
| Carnitine palmitoyltransferase 1a | ***Cpt1a*** | Forward | TTTGAATCGGCTCCTAATGG |
|  |  | Reverse | CCCAAGTATCCACAGGGTCA |
| Carnitine palmitoyltransferase 1b | ***Cpt1b*** | Forward | CAACTCCTGGAAGAAACGCC |
|  |  | Reverse | TCCACCTTGCAGTAGTTGGA |
| Fas cell surface death receptor | ***Fas*** | Forward | GGAGGTGGTGATAGCCGGTAT |
|  |  | Reverse | TGGGTAATCCATAGAGCCCAG |
| Lipase, hormone sensitive | ***Lipe*** | Forward | ATGCCACTCACCTCTGATCC |
|  |  | Reverse | CTGTCCTGTCCTTCCCGTAG |
| Lipoprotein lipase | ***Lpl*** | Forward | TGAAAGCCGGAGAGACTCAG |
|  |  | Reverse | AGTGTCAGCCAGACTTCTTCAG |
| Uncoupling protein 1 | ***Ucp1*** | Forward | ACTGCCACACCTCCAGTCATT |
|  |  | Reverse | CTTTGCCTCACTCAGGATTGG |
| Peroxisome proliferator-activated receptor alpha | ***Ppara*** | Forward | GATGTCACACAATGCAATTC |
|  |  | Reverse | CAGTTTCCGAATCTTTCAGG |
| Peroxisome proliferator-activated receptor gamma | ***Pparg*** | Forward | GCATGGTGCCTTCGCTGA |
|  |  | Reverse | TGGCATCTCTGTGTCAACCATG |
| Peroxisome proliferative-activated receptor gamma coactivator 1 alpha | ***Ppargc1a*** | Forward | AGCCGTGACCACTGACAACGAG |
|  |  | Reverse | GCTGCATGGTTCTGAGTGCTAAG |
| **qPCR conditions**: Preincubation at 95ºC for 10 min followed by 45 denaturation cycles at 94ºC for 10 s, annealing at 60ºC for 30 s and extension at 72ºC for 5 sec. | | | |

**Table S2. Mean ± standard error of the mean (SEM) of Figure 6a and Figure 6d**

| **Figure** | **mean ± SEM** | **One way ANOVA** | **Post hoc Tukey´s test (p-values)** |
| --- | --- | --- | --- |
| **Figure 6a** | CD-Veh: 2.465 ± 0.4317  HFHSD-Veh: 15.82 ± 0.4943  HFHSD-P. fae: 13.16 ± 0.8559  HFHSD-Veh p-f: 13.83 ± 0.8076 | F (3, 34) = 81.24  p <0.0001 | CD-Veh vs. HFHSD-Veh, p <0.0001 |
|  |  |  | CD-Veh vs. HFHSD-P.fae, p <0.0001 |
|  |  |  | CD-Veh vs. pair fed HFHSD, p <0.0001 |
|  |  |  | HFHSD-Veh vs. HFHSD-P.fae, p=0.0421 |
|  |  |  | HFHSD-Veh vs. pair fed HFHSD, p=0.198 |
|  |  |  | HFHSD-P.fae vs. pair fed HFHSD, p=0.898 |
| **Figure 6d**  eWAT | CD-Veh: 17.25 ± 1.263  HFHSD-Veh: 55.56 ± 2.077  HFHSD-P. fae: 46.98 ± 2.248  HFHSD-Veh p-f: 50.53 ± 2.181 | F (3, 35) = 77.80  p <0.0001 | CD-Veh vs. HFHSD-Veh, p <0.0001 |
|  |  |  | CD-Veh vs. HFHSD-P.fae, p <0.0001 |
|  |  |  | CD-Veh vs. pair fed HFHSD, p <0.0001 |
|  |  |  | HFHSD-Veh vs. HFHSD-P.fae, p=0.0184 |
|  |  |  | HFHSD-Veh vs. pair fed HFHSD, p=0.3014 |
|  |  |  | HFHSD-P.fae vs. pair fed HFHSD, p=0.5975 |
|  | CD-Veh: 14.17 ± 0.7162  HFHSD-Veh: 44.38 ± 1.456  HFHSD-P. fae: 37.10 ± 2.450  HFHSD-Veh p-f: 42.03 ± 2.139 | F (3, 34) = 64.44  p <0.0001 | CD-Veh vs. HFHSD-Veh, p <0.0001 |
|  |  |  | CD-Veh vs. HFHSD-P.fae, p <0.0001 |
| **Figure 6d**  subWAT |  |  | CD-Veh vs. pair fed HFHSD, p <0.0001 |
|  |  |  | HFHSD-Veh vs. HFHSD-P.fae, p=0.0297 |
|  |  |  | HFHSD-Veh vs. pair fed HFHSD, p=0.7819 |
|  |  |  | HFHSD-P.fae vs. pair fed HFHSD, p=0.2347 |
|  | CD-Veh: 7.686 ± 0.2293  HFHSD-Veh: 14.43 ± 0.8858  HFHSD-P. fae: 12.27 ± 0.9647  HFHSD-Veh p-f: 14.11 ± 0.7304 | F (3, 34) = 15.35  p <0.0001 | CD-Veh vs. HFHSD-Veh, p <0.0001 |
|  |  |  | CD-Veh vs. HFHSD-P.fae, p=0.0012 |
| **Figure 6d**  BAT |  |  | CD-Veh vs. pair fed HFHSD, p <0.0001 |
|  |  |  | HFHSD-Veh vs. HFHSD-P.fae, p=0.2018 |
|  |  |  | HFHSD-Veh vs. pair fed HFHSD, p=0.9912 |
|  |  |  | HFHSD-P.fae vs. pair fed HFHSD, p=0.3537 |
